# Whole Genome Sequencing Reveals Local Transmission Patterns of *Mycobacterium bovis* in Sympatric Cattle and Badger Populations

**DOI:** 10.1371/journal.ppat.1003008

**Published:** 2012-11-29

**Authors:** Roman Biek, Anthony O'Hare, David Wright, Tom Mallon, Carl McCormick, Richard J. Orton, Stanley McDowell, Hannah Trewby, Robin A. Skuce, Rowland R. Kao

**Affiliations:** 1 Boyd Orr Centre for Population and Ecosystem Health and Institute for Biodiversity, Animal Health and Comparative Medicine, University of Glasgow, Glasgow, United Kingdom; 2 Veterinary Sciences Division, Agri-Food and Biosciences Institute, Stormont, Belfast, Northern Ireland, United Kingdom; 3 School of Biological Sciences, Queen's University Belfast, Belfast, Northern Ireland, United Kingdom; University of Oxford, United Kingdom

## Abstract

Whole genome sequencing (WGS) technology holds great promise as a tool for the forensic epidemiology of bacterial pathogens. It is likely to be particularly useful for studying the transmission dynamics of an observed epidemic involving a largely unsampled ‘reservoir’ host, as for bovine tuberculosis (bTB) in British and Irish cattle and badgers. BTB is caused by *Mycobacterium bovis*, a member of the *M. tuberculosis* complex that also includes the aetiological agent for human TB. In this study, we identified a spatio-temporally linked group of 26 cattle and 4 badgers infected with the same Variable Number Tandem Repeat (VNTR) type of *M. bovis*. Single-nucleotide polymorphisms (SNPs) between sequences identified differences that were consistent with bacterial lineages being persistent on or near farms for several years, despite multiple clear whole herd tests in the interim. Comparing WGS data to mathematical models showed good correlations between genetic divergence and spatial distance, but poor correspondence to the network of cattle movements or within-herd contacts. Badger isolates showed between zero and four SNP differences from the nearest cattle isolate, providing evidence for recent transmissions between the two hosts. This is the first direct genetic evidence of *M. bovis* persistence on farms over multiple outbreaks with a continued, ongoing interaction with local badgers. However, despite unprecedented resolution, directionality of transmission cannot be inferred at this stage. Despite the often notoriously long timescales between time of infection and time of sampling for TB, our results suggest that WGS data alone can provide insights into TB epidemiology even where detailed contact data are not available, and that more extensive sampling and analysis will allow for quantification of the extent and direction of transmission between cattle and badgers.

## Author Summary

Whole genome sequencing (WGS) offers the potential for unprecedented insight into infectious diseases spread at the individual-to-individual level. However, this potential can be compromised when a poorly sampled ‘reservoir’ population contributes to transmission, as strong biases in the obtained data are inevitable. Therefore WGS data must be corroborated with epidemiological data in well-described systems, in order to enhance our confidence in their broader use. The epidemic of bovine tuberculosis (bTB) in British and Irish cattle has both economic and animal health importance; it also involves a management host (the cattle) whose demographic history is exceptionally well-documented, and with a reservoir host (the badgers) whose role in bTB spread has defied decades of study and observation. Here, we show that the observed spatial patterns provide a good match to *M. bovis* WGS data, but cattle movement networks and within-herd transmission patterns generated by mathematical models do not. Thus WGS offers considerable promise for revealing basic principles about bTB maintenance in British cattle and the role of badgers, as well as suggesting that similar approaches combining mathematical models and WGS data could be useful for the study of human TB and other infectious diseases where sampling biases are known to exist.

## Introduction

The application of whole genome sequencing (WGS) technology to infectious bacterial diseases has resulted in unprecedented advances in our ability to resolve epidemic data at the global scale [1], [2], provided new insights into within-host replication processes [3], and been used to corroborate the importance of exhaustively identified transmission chains and social drivers of transmission [4], [5]. However, evidence of its value when observing fine scale transmission dynamics in a partially sampled population is thus far limited to some tantalising observations [1] with as yet no quantitative evaluation of the underlying contact processes using nonlinear mathematical models. Such evaluations are particularly important where the sampling is biased, such as when the epidemiology involves an unobserved ‘reservoir’ host. Interpretation of sequence data at this scale is further complicated by the lag between the time of transmission and the time of sample collection, which can be considerable, especially for pathogens with extended latent periods. Comparing genetic data with mathematical models based on epidemiological contact data should allow us to develop more general transmission principles, and to compare and contrast the types of information that these different data sources provide. Combining mathematical models and pathogen sequence data has been an area of increased attention in the epidemiology of fast-evolving RNA viruses [6]–[9], but is as yet largely unexplored for bacteria, particularly for TB and other slow growing mycobacteria, where transmission intervals and routes tend to be more uncertain and evolutionary rates are slower, warranting more extensive sequence information. While this presents a unique set of challenges, WGS now offers promising research solutions to this problem.

*Mycobacterium bovis* is the causative agent of bovine tuberculosis (bTB), an important disease of both livestock and wildlife with severe socio-economic consequences and impacts on animal health. Historically, it is believed to have been a major contributor to human TB cases worldwide, and it remains a zoonotic concern in both developed and developing countries [10], [11]. While most countries that employ well developed test and slaughter programs have eliminated bTB from their livestock, the control of *M. bovis* has proven problematic in Britain and Ireland, with the Eurasian badger (*Meles meles*) implicated as an important wildlife reservoir of *M. bovis* [12]. However, despite considerable research efforts, the role of badgers in the transmission of *M. bovis* remains controversial both on scientific and socio-political grounds [13], [14].

Genotyping of *M. bovis* from cattle and badgers based on spoligotyping and Variable Number Tandem Repeat (VNTR) typing has provided considerable insight into the epidemiology of *M. bovis*. In particular, the spatial clustering of genotypes in isolates from British and Irish cattle is indicative of a locally driven transmission process [15], [16]. However, neither marker has the resolution to identify fine scale transmission patterns such as occurs at the individual herd level. Links between cattle and badgers have been identified via analysis of genotype associations and statistical analyses of observed outbreak data [17], however direct evidence of transmission chains linking the two hosts at a local farm scale remains lacking. Here, we exploit the resolution of WGS to address these questions, using samples from badgers and cattle collected from a group of neighbouring farms in Northern Ireland (NI) with a decade-long history of repeated bTB outbreaks.

The epidemic of bTB in Northern Irish cattle is particularly well described: annual ‘tuberculin’ testing of the entire cattle population creates a uniform sampling framework, and the majority of *M. bovis* isolates are now genotyped [16]. Between 2003 and 2010, this amounted to 10596 isolates from cattle, which either had a positive tuberculin test (a “reactor”) or were identified by post mortem testing of non-reactors. These data revealed 193 VNTR types within NI, with three types accounting for over 50% of sampled bacteria. *M. bovis* isolates from badgers are encountered at a lower frequency via an ongoing road traffic accident (RTA) survey, but their VNTR types show strong spatial associations on a regional scale with those found in cattle (R. Skuce, unpublished data). Complementing this extensive information on the pathogen are detailed demographic and network data: cattle locations and movements are recorded on an individual, daily basis, and retained on a centrally held database that can be cross-referenced with the *M. bovis* sample data. This combination of bacterial testing and cattle life history data provides an ideal test bed for analysing the transmission patterns reflected in WGS data.

**Figure 1:**
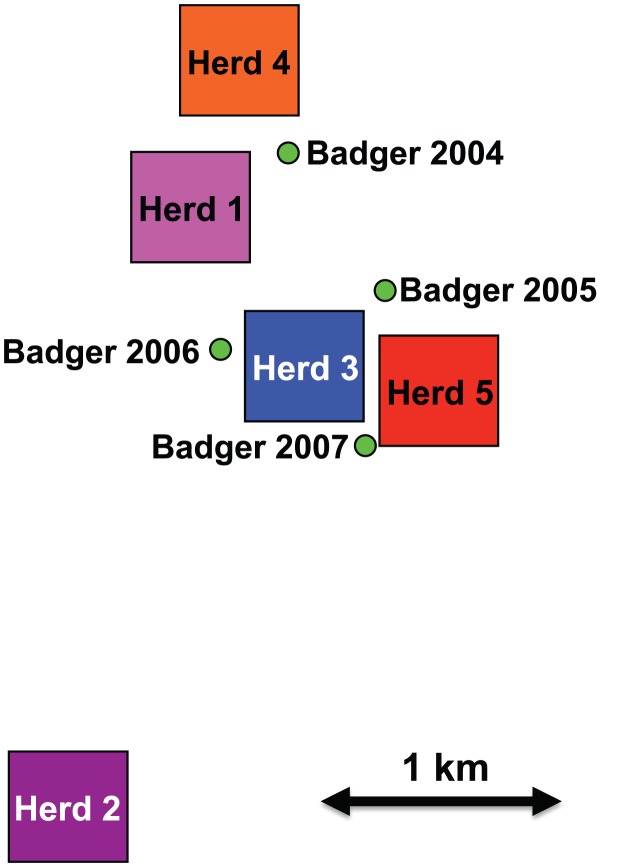
Main holdings associated with herds in the dataset and badger locations by year. Herd locations indicate centroids of main holdings, and do not include isolated land parcels or rented land.

In this study, we used whole genome sequences of *M. bovis* and mathematical modelling to analyse the transmission between and within cattle farms and the potential role of badgers in this system. A spatial cluster of five farms with recently recorded bTB breakdowns (i.e. herds with one or more reactors, or identified abattoir cases) due to VNTR type 10 was identified. This genotype is a single locus variant of VNTR type 1 (the second most common type, representing 19.2% of identified genotypes from 2003 to 2008) that is as yet of low prevalence. Type 10's relatively recent emergence means that an identifiable common source is more likely than with more broadly distributed, longer established genotypes. VNTR type 10 breakdowns had a median duration of eight months and a median of four cases each. Median breakdown size across Northern Ireland during the study period was two cases, with a median duration of seven months. Our sequence data are derived from *M. bovis* isolates from 26 cattle from the years 1999 to 2010, and 5 isolates from 4 road-kill badgers (including two from the same badger) collected from within the farm cluster between 2004 and 2007 (Figure 1).

Based on WGS and anonymised cattle data for these 31 isolates, our study aimed to i) determine the amount of genomic divergence among *M. bovis* isolates within and among herd outbreaks, including subsequent outbreaks affecting the same farm; ii) compare this to genomic diversity seen among badger isolates sampled from the same spatial area and time period as cattle; iii) test whether genetic distances among isolates correlate with either spatial distance among farms or the probability of past movement events connecting them; iv) compare the genetic results to independently obtained contact structures identified by fitting network and transmission models to the herd history data.

## Results

**Figure 2:**
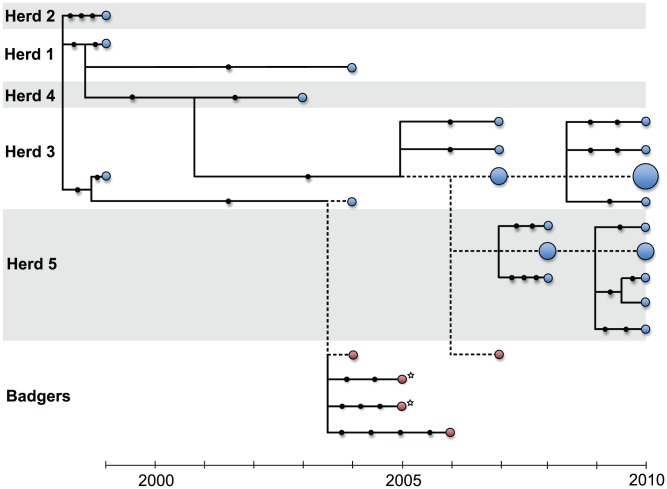
Maximum likelihood network of *M. bovis* genomes with tips arranged according to sampling date. Position of other nodes is simply shown for convenience and does not reflect known branch times. Black circles represent single nucleotide polymorphisms separating sequences, dashed lines indicates branches without mutational events. The size of the circle proportional to the number of isolates sharing the same sequence.

WGS from the 31 bTB isolates revealed a total of 39 polymorphic sites, of which 7 were shared amongst two or more isolates (Table S2 in Text S1). One of these shared polymorphisms was identified as potentially unreliable and thus excluded from the final data set (see Materials and Methods). Because *M. bovis* is believed to be clonal [15] these genome-wide data can be combined to establish the phylogenetic relationships among isolates (Figures 2 & S1). Using the known sampling dates, we found an increase in genetic divergence from the tree root through time, consistent with a molecular clock (Figure S2). The estimated evolutionary rate was 3.40 (CI: 0.87–5.93)×10^−8^ substitutions per site per year (equivalent to 0.147 substitutions per genome per year), about an order of magnitude lower than within-host mutation rates recently estimated for *M. tuberculosis* using WGS [3].

The inferred phylogeny revealed that most outbreaks involved genetically distinct isolates (Figure 2) demonstrating the suitability of WGS to track *M. bovis* spread at the herd level. The more extensively sequenced outbreaks were dominated by a single common genotype (see below). Repeated outbreaks within the same herd tended to involve closely related isolates falling into the same genetic lineage (e.g. Herd 1: 1999/2004; Herd 5: 2008/2010). The exception was Herd 3, which fit this pattern from 1999 to 2004 and from 2007 to 2010 but with distinct lineages causing outbreaks during those two periods. Though few badger isolates were available, their genetic distances from cattle isolates were small and comparable to those observed among cattle isolates; two of the five *M. bovis* genomes sequenced from badgers were genetically indistinguishable from those seen in cattle during the same year. No *M. bovis* sample taken from a badger was more than four mutational steps away from the nearest cattle isolate. Interestingly, the two isolates from different tissues of the same badger were separated by five mutational steps, as great a genetic distance as found across different cattle in serial outbreaks within the same cattle herd. This suggests either multiple infections of the same animal or a long-term infection that had allowed for within-host divergence to evolve. In contrast, isolates collected during a series of four outbreaks in Herds 3 and 5 between 2007 and 2010 showed little divergence, with 9 out of the 20 genomes being indistinguishable based on our data, despite the considerable timeframe involved.

Outbreaks could be epidemiologically linked through local transmission, which may for example involve badgers, unobserved infections in cattle, environmental contamination or contiguous contact between farms. Alternatively, transmission may be due to the network of livestock movements that connects the five farms through shared links to other farms. We compared the number of mutations separating isolates to two proxy measures of epidemiological distance, 1) the Cartesian distance between the main holding locations of cattle herds, and 2) one minus the relative probability that herds had been connected by movement of infected cattle, called here a “network separation” (see Materials and Methods for our definition of this).

**Figure 3:**
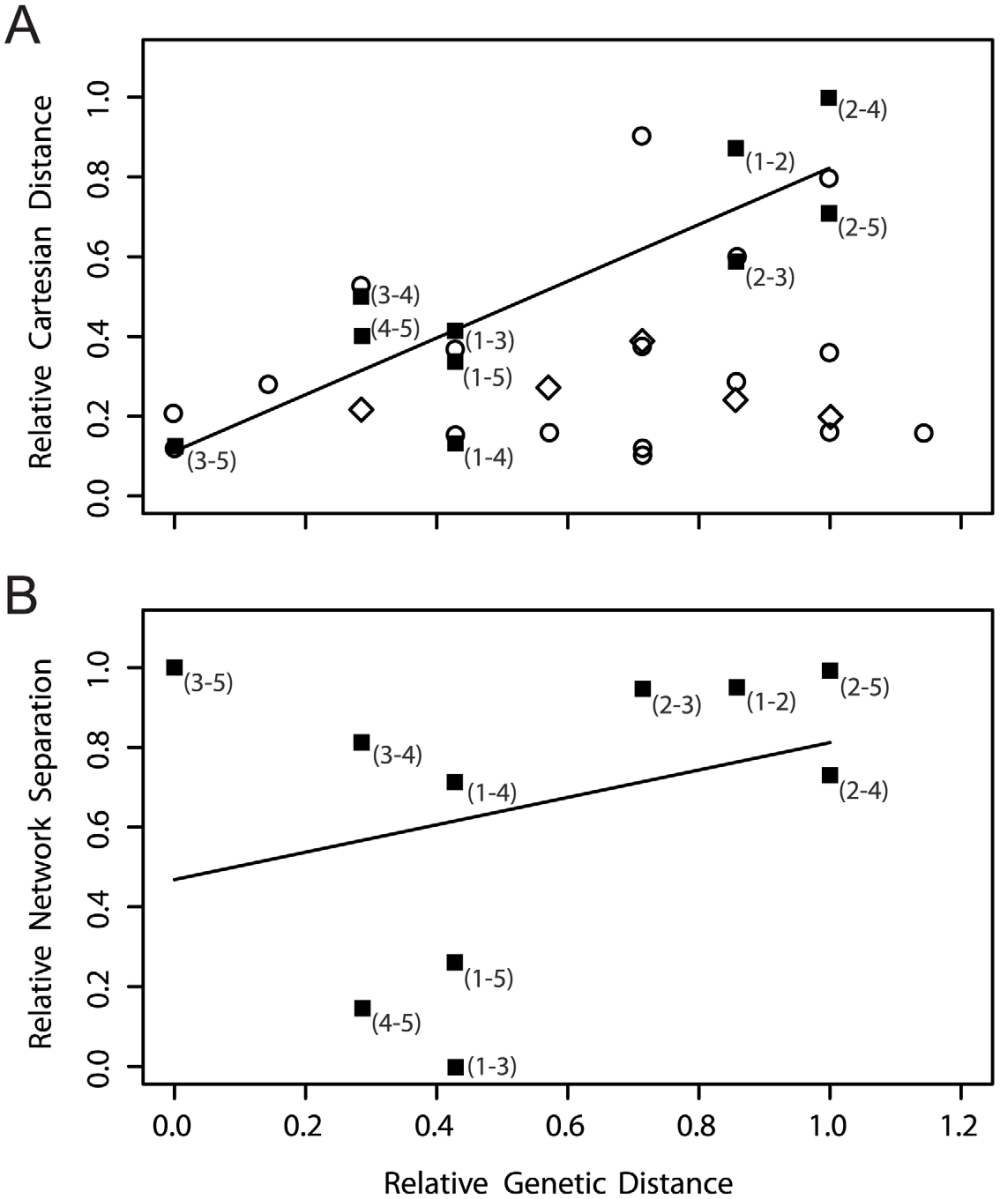
Genetic versus spatial and network distances. On both axes, all values are scaled to the maximum value for herd-to-herd interactions. On the x-axes, the minimum number of SNPs differentiating isolates from the two herds (X-Y). In panel (A) above, spatial distance versus genetic distance between herds (black squares). On the y-axis, the cartesian distance between the main holdings of two herds (X-Y) showing a high level of correlation with genetic distance (R^2^ = 0.720). For reference, the equivalent data for the badger isolates (not fitted) are shown as unfilled circles and diamonds for badger-badger and badger-herd relationships, respectively. Panel (B) below, network separation versus genetic distance between herds. On the y-axis, the network separation defined as (1−*p_ij_*), where *p_ij_* is the probability that herds *i* and *j* and linked via cattle movements through the network, considering all possible pathways through any herd from which the same genotype of M. bovis has been isolated, and p_animal_ is the per animal probability of contact that best explains the genetic distance data. The best fit value (p_animal_ = 1.35×10^−3^) shows a poor correlation with genetic distance (R^2^ = 0.094).

As there is a strong genetic and spatial autocorrelation for samples obtained from single herds, we compared only across herds, while noting that this does not completely eliminate the dependence problem. We found a positive relationship between the pair-wise Cartesian distance among farms and the smallest genetic distance among their bTB isolates (Figure 3A), consistent with at least one of the possible local transmission mechanisms being important. As would be expected, where different lineages are considered as separate outbreaks (e.g. the two lineages associated with Herd 3), there was a much poorer correlation (R^2^ = 0.444) for the earlier outbreaks compared to the later ones. To construct the cattle network, life history data were extracted for all cattle in the database from 01/01/1990 to 31/12/2010 as well as all farms in NI recording a breakdown due to VNTR type 10 between 1999 and 2010, amounting to 58 herds, 3321 cattle and 14258 recorded individual tuberculin test results from 29/07/1993 to 09/12/2010, plus records of post-mortem examination for tuberculous lesions and subsequent confirmatory tests including histology and culture. The movement network included movements amongst all herds in this group of 58 farms. The resulting network was highly connected, including our five farm cluster (Figure S3). The only two farms directly connected through recorded cattle movements were Herds 3 and 5 but this reflected a single animal and, in the absence of further indirect contact, corresponded to a high network separation. In contrast, other farm pairs were much more strongly connected through indirect contact, generally involving one or two intermediary farms. However, these reduced network separations correlated only weakly with genetic distance (Figure 3B).

**Figure 4:**
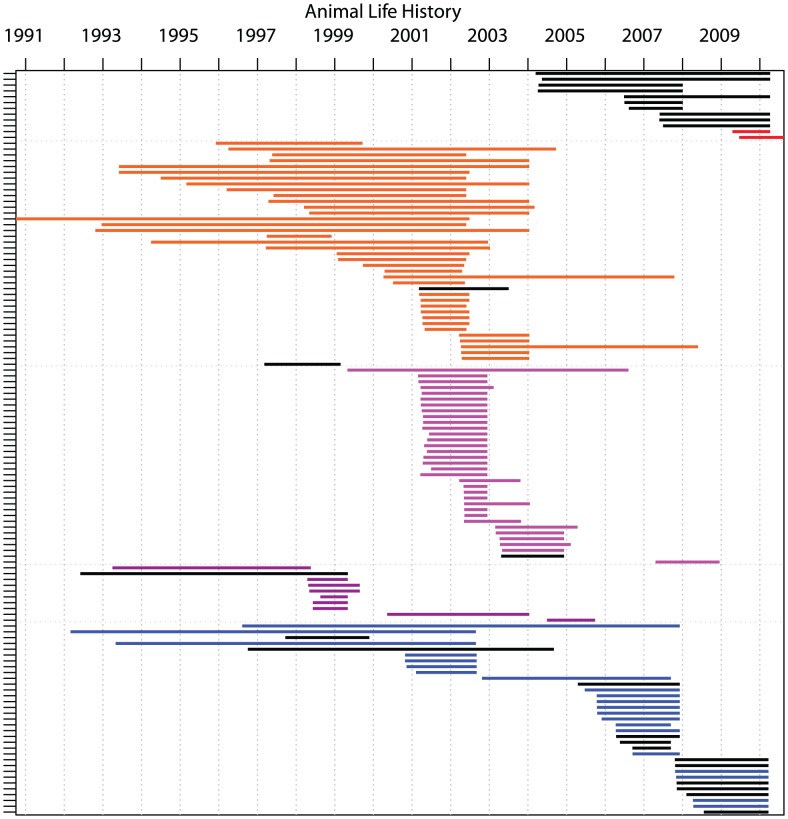
Life histories of all cattle with from which *Mycobacterium bovis* samples of VNTR type 10 were obtained. Showing all individuals residing within the five study herds at some time from 1994 to 2010. Cattle residence times indicated by the length of the horizontal bars (each bar representing a single animal). In black, all cattle from which sequences are derived (herd indicated by surrounding type). Test dates on which one herd received a whole herd test are indicated by vertical dashed lines. Herd colours correspond to colours in Figure 1 (1 – pink, 2 – purple, 3 – blue, 4 – orange, 5 – red).

Because of the indeterminate and potentially long timescales for *M. bovis* transmission, the relationship between dates of sampling and dates of transmission are uncertain. Fitting a mathematical model of transmission to the observed life history data allowed us to compare likely epidemiological processes to the observed tree structure, in order to analyse dynamics at the within-herd, individual animal level. The long period from 2007/8 to 2010 during which no reactors were identified in Herds 3 and 5, combined with the large scale depopulation that occurs following identification of a breakdown (all test reactors are slaughtered and herds are tested every two months until two consecutive clear whole herd tests are achieved), would suggest multiple introductions of infection into these herds. In contrast, the ‘flat’ genetic structure for the same outbreak, with no obvious chains, would suggest one outbreak from a single source. We therefore asked whether the recorded cattle life history and movement data, interpreted in the context of a simple nonlinear model, are consistent with the observed genetic signature. We constructed a compartmental model of transmission, assuming all animals were in one of four states: susceptible to disease (S), exposed (E), potentially test positive but not yet infectious (T) or infectious (I) [18]. Exploiting the explicit animal life histories in all five herds (Figure 4), plus import/export data from the remaining 53 herds in the group, state probability distributions for each animal were generated based on the known dates of bTB detection [6], [19]. Mechanisms other than direct transmission among identified reactors were accounted for with a single external force of infection function ‘
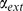
’, corresponding to the suite of possible local forces as described above, and a fitted force of infection due to latent or hidden infections, where each non-reactor is assumed to contribute to the overall force of infection in proportion to the calculated probability that itself had been infected. The overall force of infection was calculated assuming homogeneous, density dependent mixing within each herd and with contacts between herds via recorded cattle movements. A best-fit model was determined using a likelihood-based Markov Chain Monte Carlo approach (further details in the Materials and Methods section below and in Figures S4 to S6, including posterior distributions for all parameters) [20].

**Figure 5:**
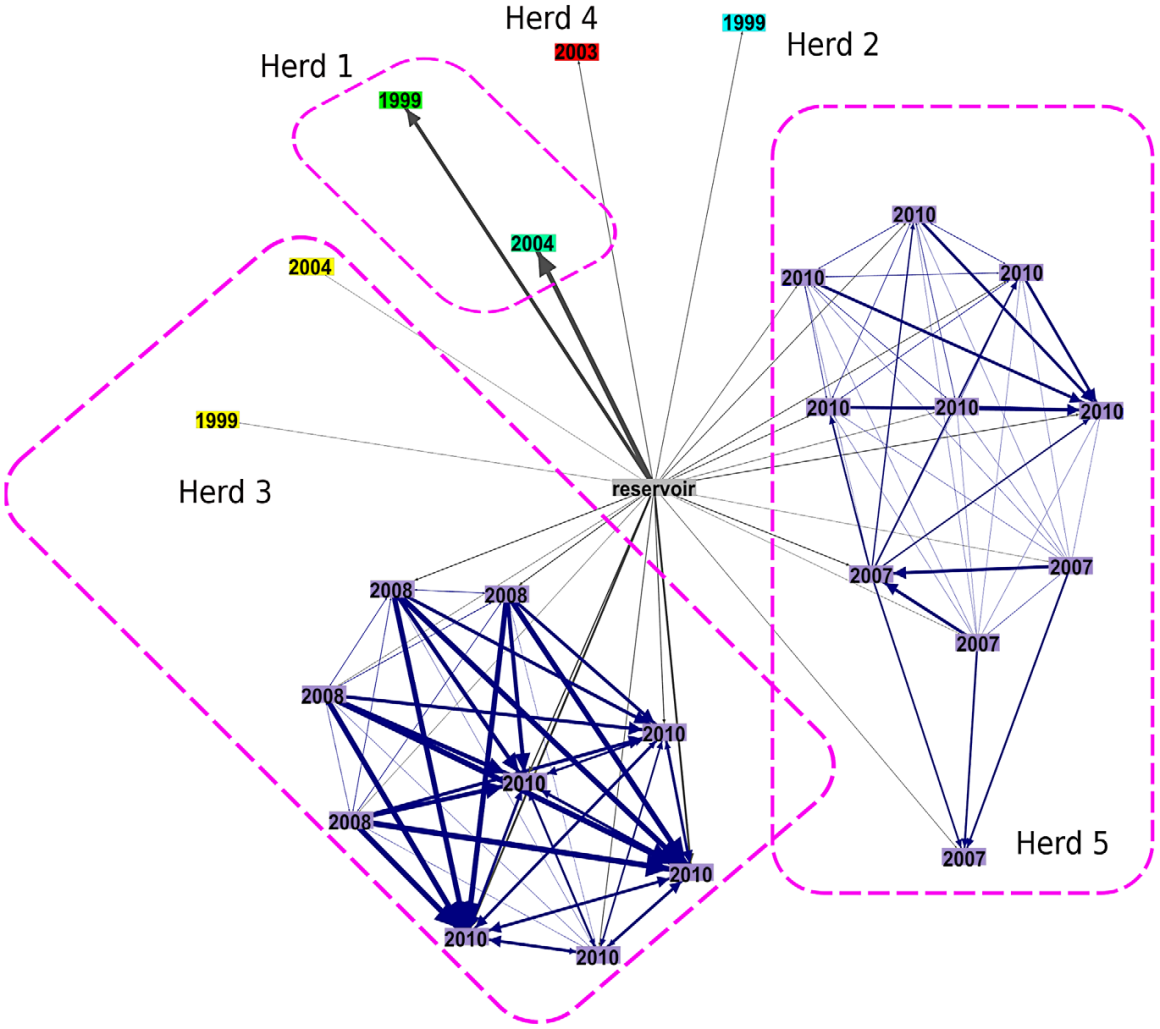
Probabilities of pairwise transmission pathways amongst infected cattle with sequenced isolates. The weighted, directed network shows the probability that a transmission path exists between cattle with sequenced isolates that does not pass through other sequenced isolates. Infection events poorly explained by transmission amongst reactor cattle are therefore more likely to be caused by a ‘reservoir’, which potentially encompasses infected badgers, between-herd interactions, latent infections, or environmental contamination. Sequences belonging to the same herd are surrounded by dashed outlines.

The considerable overlap in cattle life histories (Figure 4) could have potentially allowed for long within-herd epidemics. However, the probabilities of a transmission chain linking cattle-based isolates suggest that while cattle-to-cattle transmission can likely explain some maintenance of individual genetic lineages (at its peak, the cattle to cattle force of infection was calculated to be roughly 50× that from hidden sources), new outbreaks are usually better explained by an unspecified ‘reservoir’ (Figure 5). In particular, despite similar overlaps in reactor life histories across the entire recorded history of Herd 3, and similar observed genetic distances, the earlier outbreak is epidemiologically distinct from the later outbreak in 2007 to 2010, with separate introductions suggested for 1999 and 2004 unless at least one particular individual had an uncharacteristically long infectious period. In contrast, the 2007 to 2010 outbreaks in Herds 3 and 5 are well supported by cattle-to-cattle transmission alone. Herds 3 and 5 are the only ones recorded as having directly traded with each other, however our herd-to-herd analysis suggests that this link was unlikely to have connected the outbreaks in the two herds (Figure 3). All other herds appear only linked by the external force of infection, which was of the same order of magnitude as the force of infection due to a single infectious animal (see parameter estimates in Figure S5). Correlations between genetic distances and network distances are poor (Spearman rank correlation coefficient *r* = −0.326) with any correlation due to between- rather than within-herd differences, indicating that the WGS data are not of sufficient resolution to make inferences on transmission chains at the within-herd scale.

## Discussion

Measuring genetic variation at the whole genome scale enabled us to genetically distinguish most isolates of *M. bovis*. This is particularly notable given the small spatial extent of the study cluster, with no two farms being more than 5 km apart. Compared to traditional typing methods, for which the same genotype may be distributed over many hundred square kilometres and only broad associations can be rigorously defined [21], WGS affords a level of resolution for epidemiological studies previously limited to rapidly evolving RNA viruses [6]–[8].

In addition to most isolates and outbreaks being genetically unique we found that subsequent outbreaks on the same farm tended to involve the same genetic lineage previously detected in that location. This indicates that lineages are commonly able to persist locally within the direct environment of a farm, although the mechanisms for this are not yet clear. Results of our mathematical models based on individual cattle histories indicate that persistence on a farm is overall poorly explained by ongoing transmission within herds. In the cases of Herds 1 and 3 for example, the model identified independent introductions for subsequent outbreaks (Fig. 5), despite the fact that these serial outbreaks involved the same genetic lineage (Fig. 2). Based on these findings, the detected infections (including unsequenced reactors) are insufficient in explaining local persistence on farms, instead suggesting a number of possible alternative mechanisms, such as re-introduction of the same lineage from neighbouring herds, environmental persistence, or alternative hosts. In contrast, and despite the relative simplicity of the modelling approach used, the persistence of the outbreaks in Herds 3 and 5 from 2007 to 2010 are consistent with lineage persistence resulting from extensive within-herd transmission, despite the multiple clear whole herd tests that would have occurred in between dates for reactors. While forward simulations were used to corroborate the robustness of our modelling approach, any extrapolation of our results for bTB epidemiology in general must be viewed with caution, both because of the small size of the dataset and because some of the modelling assumptions (in particular the assumption of explicitly time dependent generation times, see supplementary information) were not intended to be mechanistic descriptions of the underlying transmission process. Nevertheless, the fact that such a parsimonious model identifies a cattle-only contact structure largely consistent with the observed phylogeny generates confidence in our results.

In addition to local persistence, we also found evidence for the introduction of new genetic lineages onto farms and our analyses allow us to partially resolve how these introductions occur. Though cattle movements are a known risk for between-herd spread of bTB in Britain [22], [23], they do not appear to be important for the events observed here, as the probabilities of transmission amongst herds involved in the extensive network of all observed VNTR10 outbreaks only poorly predict the genetic divergence amongst isolates. In contrast, we found Cartesian distance to be a good predictor of genetic distance among isolates at a very fine scale. Though the small sample size means that inferences regarding between-herd contacts should be viewed with caution, these results are consistent with the relatively low importance accorded to movements compared to local risk factors in bTB endemic areas that was previously observed at a national scale in GB [23], and suggests that a more extensive analysis of the balance between local spatial and network processes would be merited. As it stands, the most parsimonious explanation for these outbreaks involve a local transmission process that could be due to a number of causes. A non-exhaustive list of these includes both cross-boundary contact or unrecorded local movements between herds and transmission from a common badger reservoir (where the interaction is spatially localised, consistent with the badger's stable social structure and strong territoriality [24]), or a combination of these factors. While our sample size for badgers is low, the badger-derived sequences are remarkably similar to those in cattle, demonstrating very recent cross-over events between the two populations, or alternatively recent infections from a common source, such as the environment [25]. The demonstration of a high *M. bovis* diversity in a single badger suggests either a lengthy infection in that badger, or multiple exposures to different sources of infection.

Although our current estimate for the rate at which *M. bovis* genomes evolve must be considered preliminary, it is considerably slower than the rate observed in *M. tuberculosis* [3]. Should it be confirmed, this has obvious implications for the level of temporal resolution that WGS can provide for unravelling epidemiological dynamics for bTB. In our current data, we were unable to genetically resolve relationships among multiple isolates stemming from the same outbreak for example and saw serial outbreaks commonly involving identical genotypes. This is corroborated by the poor correlation between the genetic distances and our estimates of the within-herd contact structure. However, apart from limiting opportunities for molecular epidemiological inference, these observations may also hold clues with respect to *M. bovis* biology and transmission. A recent study conducting experimental infections with *M. tuberculosis* in primates, found mutation rates to be equivalent during latent and active infections and proposed oxidative damage as a potential mechanism [3]. If this is relevant to *M. bovis*, one could hypothesise that the slower rates of evolution seen here at the population level, could be caused by the bacterium spending extended periods outside the host, in the environment. Future studies and analyses are needed to obtain more accurate estimates for the genomic rate of evolution in *M. bovis* and to test for potential rate heterogeneity and its underlying factors.

While cattle movements and long-term, hidden persistence within herds have both been shown to contribute significantly to herd breakdowns [22], [23], [26], these previous analyses were aimed at the identification of statistical associations; here we have shown that WGS data are able to identify local interactions as the principle culprit in specific herds. This makes WGS both a valuable tool for forensic epidemiology, and an aid in the construction of improved mathematical and statistical models of disease dynamics. In contrast, the poor correlation between network and genetic distance at the within-herd level suggests important limits to the resolution that WGS can provide for this system. The local effects identified here may be due to the local infected badger population, but are also consistent with local herd- to-herd spread. Our simplified modelling approach was chosen to maximise the use of available epidemiological contact data, but at the expense of a more detailed exploration of the possible hypotheses regarding the sources of transmission. However, it is likely that WGS based on more extensive sampling will allow for more sophisticated approaches, that could be used to directly estimate the role of badgers in the maintenance of bTB in British and Irish cattle. While insights into particular disease problems will depend on many factors we cannot consider here, our study supports the proposition that WGS data alone can provide insight into the impacts of unobserved populations on observed epidemics even in the absence of detailed transmission chain information, for *M. bovis*, other members of the *M. tuberculosis* complex, and other pathogens involving reservoir hosts.

## Materials and Methods

### VNTR typing

*M. bovis* is a member of the closely related *Mycobacterium tuberculosis* complex, which consists of several species with a shared ancestry [27] but which have evolved marked but not absolute host preferences [28], [29]. On a global scale, the *M. tuberculosis* complex can now be subdivided into discrete lineages, which show strong phylogeographical localisation to regions [30], [31]. VNTR profiling is a genotyping technique based on determining the copy number of a series of short, simple DNA repeats, originally identified by genome analysis [32]. However, while mutations in VNTR type have been observed within the timescale of observation at the regional level, in most cases, probable transmission events are associated with the same VNTR-type, therefore requiring finer resolution typing to order the members of these groups.

Herd-level *M. bovis* genotyping has been performed by the Agri-Food and Biosciences Institute, Belfast, UK since 2003, as follows. The first (disclosing) *M. bovis* isolate from all bovine TB herd incidents was subjected to genotyping (eight-VNTRs and spoligotyping convention). Heat-inactivated *M. bovis* cell lysates were used directly as PCR-ready templates. VNTR profiling, spoligotyping, nomenclature, reference strains and quality control were as described [32]. The inferred tandem repeat copy number at each VNTR locus was used to produce a concatenated multi-locus VNTR profile (a string of integers), which was then simplified to a number indicating the prevalence of that profile. Genotype 001 (SB0140), with a spoligotype of SB0140, was the most prevalent in Northern Ireland when surveyed in 1999 to 2003 [32]. Spoligotypes were named according to an agreed international convention (www.mbovis.org).

### Datasets

Anonymised records of cattle tuberculin tests, farm locations and cattle movements among them were drawn from the Animal and Public Health Information System (APHIS), a database containing details of all cattle in Northern Ireland that is administered by the Department of Agriculture and Rural Development [33]. The locations (main farm building) of 58 herds that had a breakdown with VNTR type 10 between 1999 and 2010 were extracted from APHIS. A cluster of five of these herds was selected based on their spatial clustering and the proximity of badger-derived isolates of type 10. The life histories of all 3299 cattle that passed through them since 1995 were compiled, comprising birth and death dates, and the dates of movements into and out of the cluster herds. These animals underwent routine bTB skin testing every year and the lifetime test history of each animal was extracted, containing the dates and results of all tuberculin tests (a total of 14258 individual test results). Results of post-mortem examination for tuberculous lesions and any subsequent confirmatory tests (laboratory based histology, culture and VNTR typing) were also incorporated.

Cattle movement was investigated by extracting a broader dataset, comprising all herds that animals passing through the 58 VNTR 10 herds had also visited (a total of 14 096 herds, excluding livestock markets). All cattle movements among the expanded set between 1992 and 2010 were extracted, a total of 5,875,510 individual animal movements.

### DNA extraction and sequence analysis

*M. bovis* was isolated and confirmed from suspect bovine granulomatous tissue using standard protocols. Confirmed cultures were grown to single colonies on LJ slopes and single colonies were amplified for DNA extraction using the standard CTAB and solvent extraction protocol [34]. Extracted DNA was sequenced at the Sir Henry Wellcome Functional Genomics Facility at the University of Glasgow using an Illumina Genome Analyser IIx. Pair-end reads of 70 bp in length, separated by an average of about 350 bp, were trimmed from both ends based on phred quality scores so as to result in an error rate of 0.001 or less for each base call in the remaining sequence. Reads were mapped to a published UK reference genome (AF2122/97) [35] using the Geneious assembler under the “medium-low sensitivity” option, allowing for a maximum of 10% gaps and mismatches per read [36]. The reference sequence belongs to the same spoligotype (SB0140) as VNTR type 10 and shares identical repeat numbers with it for four out of eight loci used for typing. Mapping resulted in greater than 99% genome coverage with at least 1× and an average read depth of 60–112× for all isolates (see Table S1 in Text S1 for full details). Consensus sequences were generated from the mapped contig based on the quality score sum for each position. A cumulative quality score threshold of 60 (corresponding to an error probability of 1 in 1,000,000) was applied to each position to ensure that accuracy of the final consensus sequence was dependent on both quality and read depth, rather than read depth alone. Below this threshold, the consensus base call was scored as unknown (“N”). Alignment of consensus sequences was carried out using Mauve [37], as implemented within Geneious, assuming collinear genomes and with automatic calculation of seed weight and of the minimum Locally Collinear Blocks (LCB) score. Regions that were difficult to align or which contained >3 consecutive columns of unknown bases or gaps were removed from the final alignment. Similarly, sites that were polymorphic solely due to one or more sequences having ambiguity base calls were removed; this was the only context in which ambiguities were observed. The final alignment, which still represented 99.2% of the reference genome, thus only contained dimorphic single site polymorphisms situated within otherwise invariable regions.

After stripping identical sites, a total of 39 SNPs were identified (38 substitutions, 1 deletion, Table S2 in Text S1), of which seven were shared between two or more sequences. All SNPs were examined to confirm their validity before further analysis. Of particular concern was the potential inclusion of spurious SNPs associated with repeat regions in the genome for which mapping may be unreliable. While four of the SNPs were found to fall either in or close to potentially problematic regions, the reliability of the mapping and SNP calling could be confirmed in all four cases (see Supplementary Materials). All SNP calls were supported by at least 38× coverage, with high consistency among reads (usually>95%). The only exception to this was a SNP in position 221927 (G−>A), for which consensus calling was ambiguous in one of the four isolates in which it occurred (Herd5_E_2010, 92×, A: 64%, G: 36%, Table S2 in Text S1). Preliminary analyses further showed that the phylogenetic information provided by this site was in conflict with that of other informative positions (which were in complete agreement). Because these observations raised doubts about the reliability of scoring this SNP as well as about the information it provided, the site was removed from the data set. The final data set was used to generate a maximum likelihood tree using phyml [38] under a Jukes-Cantor model using a heuristic search and the reference genome for outgroup rooting.

All sequence data generated for this project are available from the European Nucleotide Archive (http://www.ebi.ac.uk/ena/) under accession number ERP001418.

### Modelling of transmission chains

Using the anonymised life history data of the herds containing genotyped isolates, we adapted a method previously used to study foot and mouth disease virus epidemiology and transmission phylodynamics at the between-farm scale [6], [19]. All cattle were assumed to be in one of four states that reflect the critical points in the cattle infection process [18], [39]–[41]: susceptible (S), exposed (E), where an animal is infected but neither tests positive nor infects others, potentially test positive (T), where the animal can test positive but is not yet infectious and infectious (I), with a constant transition rate from the E to T states and from the T to I states. States T or I can be truncated by a positive test. Knowing the dates each reactor (an animal that tests positive) was detected, we use the transition rates to determine the set of distribution functions for each animal describing the probability of being in each of the four infection states at any time in its life. We used these distributions to determine the likelihood of the observed pattern of reactors in the cluster of herds.

This key simplification (conditioning on the observed test data) is less rigorous than an approach where the likelihood is integrated forward across all possible dates of infection and the observed data is treated as a random variable. However the latter approach would have been computationally prohibitive for our system. The simplification implies that mean generation times are consistent over the observed timeframe, even though they would naturally be expected to contract over the course of an epidemic [42]. The assumption is expected to be reasonable if the disease is in an endemic state, or an endemic state is rapidly reached after the initial introduction, where ‘rapid’ is relative to the observed timescale – i.e. the distribution across states is ‘quasi-stationary’, with E and T proportional to the reciprocal of their respective transition rates, i.e. 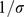 and 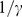 respectively. Though observation of our incidence data suggested this to be likely, to test this assumption, we allowed the proportion in state I at the time of detection to vary over the course of the epidemic, with probability 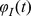 that it is infectious (i.e. in the I state) and 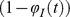 for being in the T state, where *t* is the time since the genotype was initially detected. Here, we have assumed that 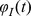 has the form 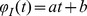, where *a* and *b* are fitted constants and 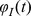 is a probability with range [0…1].

We assumed that all cattle are equally infectious if in state ‘I’ but that an infected animal does not contribute to further infection after it is detected; most reactor cattle are removed from the herd within days of being identified. All infection is caused by either infectious cattle or by a reservoir (either undetected infected cattle within the herd or external, probably local, factors).

At this population scale, we assumed that the probability of a false positive is negligible. To account for untested and undetected contributions to the epidemic, we assumed all test-negative/untested cattle to have a fitted probability of being infectious.

The date(s) of an animal “*i*” having a positive test is denoted as 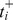, at which it may be infectious with a probability 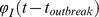, where 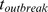 is the time the outbreak was initially detected in the herd and *t* is equal to 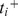 at the point of evaluation. We assumed that a reactor in the infectious state became infectious (i.e. moved from the test sensitive state) within a maximum of 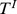 days prior to the positive test. 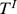 is the maximum infectious lifetime of the animal prior to detection. Because a uniform test is applied to all cattle over 6 months of age on an annual basis, the date of first test after infection would fall in the range of 0 to 1 year; therefore we assume that the duration of the effective infectious period is uniform. This has the (conservative) effect of minimising the potential duration of cattle-only transmission chains since it does not allow for long infectious periods. The probability that a reactor was infectious at any time prior to the date it tested positive is:
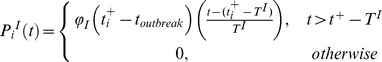
All other model states were assumed to be exponentially distributed. The probability that an individual is test sensitive at a given time *t* is therefore
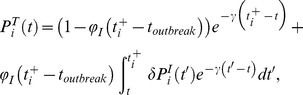
where 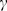 is the transition rate from the T to the I states in the forward SETI model. The parameter 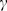 determines the duration of the state T and therefore the time when the backwards T to E transition occurs, conditional on the backwards transition from I to T and the time of detection 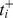. The first term accounts for the animal being in the potentially test positive state at 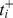 and the second term accounts for the animal being infectious at 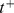. Here, 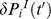 is the increase in 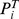 that occurs due to transition from 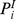 at time *t′*. Similarly the probabilities of being in the exposed and susceptible states are
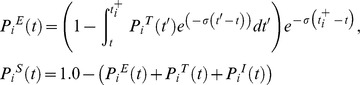
where 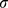 is the transition rate from the E to the T states in the forward-in-time model. The forward-in-time transition from the S to the E state is determined by 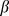 and is estimated via the calculated force of infection based on the probabilities of being in the I state. It is incorporated directly into the calculation of the likelihood as described below.

A similar approach was used for animals identified by a post mortem test in which case 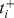 is the date the animal was post mortem examined. We assumed further that post mortem testing only identifies the same infection classes as the standard tuberculin test (i.e. only *T* or *I* class individuals are detected).

We calculated the probability that an animal became infected by considering the forces of infection on it during its lifetime. The life history of each animal that resided in the cluster between January 1990 and 31 December 2010 was converted into a lookup table of the herds in which it resided in fortnightly time steps. If an animal moved between herds in a time step it was treated as belonging to both herds since it can contribute to an outbreak in both herds during that step. Animals born into a herd are added to the lookup table for that time step and conversely death results in an animal being removed from the lookup table – movements back to the herd cluster via other herds are also retained. Using this lookup table we were able to calculate the probability an animal became infected in each period.

We calculate the probability that an animal would have become infected at any time *t*, 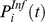, given that is was a known reactor based on the force of infection calculated from the model. If an animal was a reactor, then the incremental probability 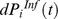 that an animal, *i*, was infected by all sources over the time interval *t*, *t+dt* is therefore given as
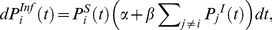
(1)calculated using the explicit infection histories. If the animal never tested positive then
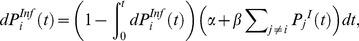
(2)i.e. we consider the probability that an animal was infected despite never testing positive. Young calves destined for slaughter at a young age are test exempt and are considered to be a negligible risk due to their short lifespan; they are therefore excluded from the analysis. The parameter 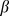 is the transmission rate from a contact with an infected animal in the same herd at the same time (the summation is over animals in the same herd at *t*). The reservoir term, 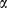, can be written as 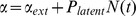 i.e. a combination of any external forces of infection, 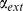, (e.g. infected wildlife) that is represented by a fixed rate and 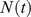, the fraction of herd at *t* that never tested positive, for which the contribution of latent infections is summarized by the probability *P_latent_*.

We calculated the probability each animal was infected during its lifetime by integrating 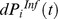 over the animals' entire life (approximated by a summation over fortnightly time steps) and thus calculate the likelihood for the model as:
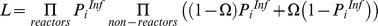
Here, 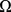 is the fitted sensitivity of the test for bTB infection. Consistent with the low number of reactors in officially TB free countries such as Scotland, the test specificity is assumed to be effectively 100%. Using *L*, we used the Metropolis-Hastings algorithm to derive the posterior distributions over the parameter space defined by 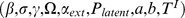. The length of burn-in for the MCMC simulations was checked using multiple MCMC chains with dispersed starting points, perturbing a subset of the parameters at each step in the chain (further descriptions in the supplementary information, and Figures S4 and S7 for the evolution of the MCMC chains and the posterior distribution of the parameters). Prior distributions were chosen to be uniform, with ranges 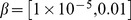, 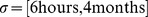, 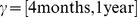, 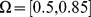, 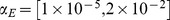, 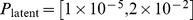, 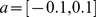, 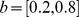 and 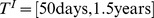, based on the parameters estimates previously reported in various field and experimental studies [43]–[45].

A running sum of the probability of a transmission event (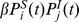) was retained, ignoring potential transmissions that were incompatible with the phylogenetic tree (for example between animals with isolates in different clades of the tree, excluding these animals from being linked by transmission). We used a modified Dijkstra's algorithm [46] to identify all possible infection paths between cattle for which we have isolates. Each link “*i*” in a defined chain has a probability *p_i_* of being associated with transmission, so that the probability of that transmission chain occurring is given by the product of the *p_i_'s*. Then the probability that a path will exist between two individuals *A* and *B* is simply the probability that at least one of the identified possible chains will connect the two, and therefore
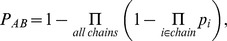
The calculation included all chains that do not pass through another sequenced isolate, and for computational convenience, we have limited the calculation to chains of less than three individual intermediate cattle. In order to test our assumptions, we ran forward simulations of a SETI model, based on the most likely parameter values of the posterior distributions from the outbreak data analysis. Applying our estimation approach to the simulated outbreak data confirmed that all the input parameter values fell within the 95% credible intervals of their corresponding posterior distributions derived from the original analysis of the data (not shown).

### Identifying the best-fit network distance between herds

Similar to the approach described above, all paths amongst Herds 1 to 5 that do not pass through the remaining herds were calculated. The link strength probability between Herds C and D was taken to be
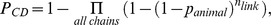
where the product considers any direct path (only between Herds 3 and 5), all chains of path length 2 (i.e. with one intermediate), or where these do not exist, chains of path length 3. The parameter *n_link_* is the number of cattle moved between pairs of premises in the chains. The ‘network separation’, defined as {1−*P_CD_*}, was used as a measure of within-herd network distance, and this was plotted against the minimum genetic distance between the two herds. The value of *p_animal_* (i.e. the relative weighting assigned to each animal moved, where 0<*p_animal_*<1) was set to optimise the R^2^ value (Figure 3B).

## Supporting Information

Figure S1 **Maximum likelihood tree of 31** ***M. bovis*** **genomes based on 38 concatenated SNPs.** Tree search was conducted in phyml under a Juke-Cantor model and reference sequence AF2122/97 was used as an outgroup (removed for clarity) to establish the root node.
(EPS)

Figure S2 **Accumulation of genetic changes in** ***M. bovis*** **genomes through time.** Genetic divergence of 31 cattle and badger isolates from the root node (measured in substitutions per genome) increases with sampling date, consistent with clock-like evolution. The estimated slope corresponds to an evolutionary rate of 3.40 (CI: 0.87–5.93)×10^−8^ substitutions per site per year. To improve visibility, points were jittered randomly.
(EPS)

Figure S3 **Network of contact via cattle movements amongst all cattle herds in Northern Ireland where breakdowns due to VNTR type 10 have been identified.** Herds from which sequenced isolates were derived are indicated in red and number as in Figure 1. All other herds in yellow.
(TIF)

Figure S4 **Evolution of the Gelman-Rubin shrink factor for the Markov Chain Monte Carlo chains (burn-in period removed).** Each chain (6 in total) was started at a different point in the parameter space of the model. At each step in the chain we perturbed the set of parameters to make the next step and if accepted, calculate the log-likelihood for the model. The potential scale reduction factor is calculated as <1.01 after a long burn in phase indicating convergence has been reached.
(TIF)

Figure S5
**Posterior kernel density estimates.** Illustrated are the distributions obtained from the MCMC chains after the burn-in period was removed. Convergence towards optimum values in all parameters is observed, with considerable mixing of the parameters. Here 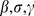 are the transition rates from the susceptible to exposed, exposed to test sensitive and test sensitive to infectious states respectively, 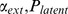 are the external and internal (latently infected animals within the herd) reservoir terms respectively, T^I^ is the length of the infectious stage and *a* and *b* inform the probability 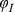 that a reactor animal was infectious (rather than test sensitive) at the time of a positive test, according to the form 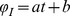. Mean posterior values with 95% credible intervals (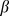 = 3.52 [2.27, 4.77]×10^−3^ fortnight^−1^ 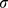 = 0.387 [0.382, 0.392] fortnight^−1^, 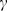 = 0.266 [0.262, 0.270] fortnight^−1^, 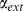 = 0.633 [0.631, 0.635] fortnight^−1^, *P_latent_* = 1.010 [0.600, 1.430]×10^−4^ fortnight^−1^, *a* = 0, *b* = 0.0860 [0.0837, 0.0884], *T^I^* = 71.6 [68.9, 74.3] days. See **Figure S6** for the distribution of all the points sampled.(PS)

Figure S6
**Distribution of the samples taken in the Markov Chain.** The lower panel shows all the sampled points of the Markov Chain and the upper is colour coded with the lighter colours denoting those samples corresponding to a higher likelihood. The clumping that is observed in the sampling regime for some parameters is due to the convergence of the chains. Here 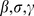 are the transition rates from the susceptible to exposed, exposed to test sensitive and test sensitive to infectious states respectively, 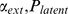 are the external and internal (latently infected animals within the herd) reservoir terms respectively, T^I^ is the length of the infectious stage and *a* and *b* inform the probability 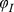 that a reactor animal was infectious (rather than test sensitive) at the time of a positive test, according to the form 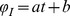, The priors used can be seen from the limits of the sampled points, in each case we used uniform priors over these limits. The parameter *L* is the maximum length of the infectious period (in days). The priors used for 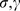 correspond to stages with lengths 1–110 days and 120–280 days respectively, and for 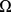, 0.50–0.85.(PNG)

Figure S7
**Trace of the parameters of the model.** Illustrated are the traces of the parameters used in the model. Convergence towards posterior values in all parameters is observed from the dispersed starting points. Here 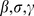 are the transition rates from the susceptible to exposed, exposed to test sensitive and test sensitive to infectious states respectively, 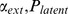 are the external and internal (latently infected animals within the herd) reservoir terms respectively, T^I^ is the length of the infectious stage and *a* and *b* inform the probability 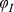 that a reactor animal was infectious (rather than test sensitive) at the time of a positive test, according to the form 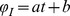, 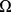 is the sensitivity of the routine herd test applied to each animal.(PNG)

Text S1 **Supporting Material and Methods: Examining the reliability of SNP calls among the 31 VNTR-10 isolates.**
(DOC)

## References

[1] Harris SR, Feil EJ, Holden MTG, Quail MA, Nickerson EK, et al (2010) Evolution of MRSA During Hospital Transmission and Intercontinental Spread. Science 327: 469–474. 20093474 10.1126/science.1182395 PMC2821690

[2] Morelli G, Song YJ, Mazzoni CJ, Eppinger M, Roumagnac P, et al (2010) *Yersinia pestis* genome sequencing identifies patterns of global phylogenetic diversity. Nat Genet 42: 1140–3. 21037571 10.1038/ng.705 PMC2999892

[3] Ford CB, Lin PL, Chase MR, Shah RR, Iartchouk O, et al (2011) Use of whole genome sequencing to estimate the mutation rate of *Mycobacterium tuberculosis* during latent infection. Nat Genet 43: 482–486. 21516081 10.1038/ng.811 PMC3101871

[4] Schürcha AC, Kremer K, Kiers A, Davienaa O, Boereec MJ, et al (2010) The tempo and mode of molecular evolution of *Mycobacterium tuberculosis* at patient-to-patient scale. Infect Genet Evol 10: 108–114. 19835997 10.1016/j.meegid.2009.10.002

[5] Gardy JL, Johnston JC, Sui SJH, Cook VJ, Shah LN, et al (2011) Whole-Genome Sequencing and Social-Network Analysis of a Tuberculosis Outbreak. N Engl J Med 364: 730–739. 21345102 10.1056/NEJMoa1003176

[6] Cottam EM, Thebaud G, Wadsworth J, Gloster J, Mansley L, et al (2008) Integrating genetic and epidemiological data to determine transmission pathways of foot-and-mouth disease virus. Proc R Soc Lond B Biol Sci 275: 887–895. 10.1098/rspb.2007.1442 PMC2599933 18230598

[7] Koelle K, Cobey S, Grenfell B, Pascual M (2006) Epochal evolution shapes the phylodynamics of interpandemic influenza A (H3N2) in humans. Science 314: 1898–1903. 17185596 10.1126/science.1132745

[8] Volz EM, Pond SLK, Ward MJ, Brown AJL, Frost SDW (2009) Phylodynamics of Infectious Disease Epidemics. Genetics 183: 1421–1430. 19797047 10.1534/genetics.109.106021 PMC2787429

[9] Holmes EC, Grenfell BT (2009) Discovering the phylodynamics of RNA viruses. PLoS Comput Biol 5: e1000505. 19855824 10.1371/journal.pcbi.1000505 PMC2756585

[10] Cosivi O, Grange JM, Daborn CJ, Raviglione MC, Fujikura T, et al (1998) Zoonotic tuberculosis due to *Mycobacterium bovis* in developing countries. Emerg Infect Dis 4: 59–70. 9452399 10.3201/eid0401.980108 PMC2627667

[11] Evans JT, Smith EG, Banerjee A, Smith RM, Dale J, et al (2007) Cluster of human tuberculosis caused by *Mycobacterium bovis*: evidence for person-to-person transmission in the UK. Lancet 369: 1270–1276. 17434402 10.1016/S0140-6736(07)60598-4

[12] Bourne J, Donelly CA, Cox DR, Gettinby G, McInerney JP, et al (2000) Bovine tuberculosis: towards a future control strategy. Vet Rec 146: 207–210. 10731068

[13] Anon (2010) The killing fields. Nature 467: 368–368. 20864950 10.1038/467368a

[14] Spencer A (2011) One body of evidence, three different policies: bovine tuberculosis policy in Britain. Politics 31: 91–99.

[15] Smith NH, Gordon SV, de la Rua-Domenech R, Clifton-Hadley RS, Hewinson RG (2006) Bottlenecks and broomsticks: the molecular evolution of *Mycobacterium bovis* . Nat Rev Microbiol 4: 670–681. 16912712 10.1038/nrmicro1472

[16] Skuce RA, Mallon TR, McCormick CM, McBride SH, Clarke G, et al (2010) *Mycobacterium bovis* genotypes in Northern Ireland: herd-level surveillance (2003 to 2008). Vet Rec 167: 684–689. 21257483 10.1136/vr.c5108

[17] Woodroffe R, Donnelly CA, Cox DR, Gilks P, Jenkins HE, et al (2009) Bovine Tuberculosis in Cattle and Badgers in Localized Culling Areas. J Wildl Dis 45: 128–143. 19204342 10.7589/0090-3558-45.1.128

[18] Kao RR, Roberts MG, Ryan TJ (1997) A model of bovine tuberculosis control in domesticated cattle herds. Proc R Soc Lond B Biol Sci 264: 1069–1076. 10.1098/rspb.1997.0148 PMC1688544 9263472

[19] Haydon DT, Woolhouse ME, Kitching RP (1997) An analysis of foot-and-mouth-disease epidemics in the UK. IMA J Math Appl Med Biol 14: 1–9. 9080685

[20] Hastings WK (1970) Monte Carlo Sampling Methods Using Markov Chains and Their Applications. Biometrika 57: 97–109.

[21] Monaghan ML, Doherty ML, Collins JD, Kazda JF, Quinn PJ (1994) The Tuberculin Test. Vet Microbiol 40: 111–124. 8073619 10.1016/0378-1135(94)90050-7

[22] Gilbert M, Mitchell A, Bourn D, Mawdsley J, Clifton-Hadley R, et al (2005) Cattle movements and bovine tuberculosis in Great Britain. Nature 435: 491–496. 15917808 10.1038/nature03548

[23] Green DM, Kiss IZ, Mitchell AP, Kao RR (2008) Estimates for local and movement-based transmission of bovine tuberculosis in British cattle. Proc R Soc Lond B Biol Sci 275: 1001–1005. 10.1098/rspb.2007.1601 PMC2366193 18252669

[24] Roper TJ (2010) Badger. London: Harper Collins.

[25] Courtenay O, Reilly LA, Sweeney FP, Hibberd V, Bryan S, et al (2006) Is Mycobacterium bovis in the environment important for the persistence of bovine tuberculosis? Biol Lett 2: 460–462. 17148430 10.1098/rsbl.2006.0468 PMC1686208

[26] Karolemeas K, McKinley TJ, Clifton-Hadley RS, Goodchild AV, Mitchell A, et al (2011) Recurrence of bovine tuberculosis breakdowns in Great Britain: Risk factors and prediction. Prev Vet Med 102: 22–29. 21767886 10.1016/j.prevetmed.2011.06.004

[27] Brosch R, Gordon SV, Marmiesse M, Brodin P, Buchrieser C, et al (2002) A new evolutionary scenario for the Mycobacterium tuberculosis complex. Proc Natl Acad Sci USA 99: 3684–3689. 11891304 10.1073/pnas.052548299 PMC122584

[28] Smith NH, Kremer K, Inwald J, Dale J, Driscoll JR, et al (2006) Ecotypes of the Mycobacterium tuberculosis complex. J Theor Biol 239: 220–225. 16242724 10.1016/j.jtbi.2005.08.036

[29] Wirth T, Hildebrand F, Allix-Beguec C, Wolbeling F, Kubica T, et al (2008) Origin, spread and demography of the *Mycobacterium tuberculosis* complex. PLoS Pathog 4 9 e1000160. 18802459 10.1371/journal.ppat.1000160 PMC2528947

[30] Gagneux S, Small PM (2007) Global phylogeography of *Mycobacterium tuberculosis* and implications for tuberculosis product development. Lancet Infect Dis 7: 328–337. 17448936 10.1016/S1473-3099(07)70108-1

[31] Hershberg R, Lipatov M, Small PM, Sheffer H, Niemann S, et al (2008) High functional diversity in *Mycobacterium tuberculosis* driven by genetic drift and human demography. PLoS Biol 6: 2658–2671. 10.1371/journal.pbio.0060311 PMC2602723 19090620

[32] Skuce RA, McDowell SW, Mallon TR, Luke B, Breadon EL, et al (2005) Discrimination of isolates of *Mycobacterium bovis* in Northern Ireland on the basis of variable numbers of tandem repeats (VNTRS). Vet Rec 157: 501–504. 16244231 10.1136/vr.157.17.501

[33] Houston R (2001) A computerised database system for bovine traceability. Rev Sci Tech 20: 652–661. 11548534 10.20506/rst.20.2.1293

[34] Van Soolingen D, de Hass P, Kremer K (2002) Restriction fragment length polymorphism (RFLP) typing of Mycobacteria. Bilthoven: National Institute of Public Health and the Environment.

[35] Garnier T, Eiglmeier K, Camus JC, Medina N, Mansoor H, et al (2003) The complete genome sequence of *Mycobacterium bovis* . Proc Natl Acad Sci USA 100: 7877–7882. 12788972 10.1073/pnas.1130426100 PMC164681

[36] Drummond AJ, Ashton B, Buxton S, Cheung M, Cooper A, et al. (2010) Geneious v5.3, Available from http://www.geneious.com.

[37] Darling AC, Mau B, Blattner FR, Perna NT (2004) Mauve: multiple alignment of conserved genomic sequence with rearrangements. Genome Res 14: 1394–1403. 15231754 10.1101/gr.2289704 PMC442156

[38] Guindon S, Gascuel O (2003) A simple, fast and accurate algorithm to estimate large phylogenies by maximum likelihood. Syst Biol 52696–52704. 10.1080/10635150390235520 14530136

[39] Barlow ND, Kean JM, Hickling G, Livingstone PG, Robson AB (1997) A simulation model for the spread of bovine tuberculosis within New Zealand cattle herds. Prev Vet Med 32: 57–75 doi: 10.1016/S0167-5877(97)00002-0 9361321 10.1016/s0167-5877(97)00002-0

[40] Agusto FB, Lenhart S, Gumel AB, Odoi A (2011) Mathematical analysis of a model for the transmission dynamics of bovine tuberculosis. Math Meth Appl Sci 34: 1873–1887 DOI: 10.1002/mma.1486.

[41] Goodchild AV, Clifton-Hadley RS (2001) Cattle-to-cattle transmission of *Mycobacterium bovis* . Tuberculosis 81: 23–41 DOI: 10.1054/tube.2000.025641. 11463222 10.1054/tube.2000.0256

[42] Kenah E, Lipsitch M, Robins JM (2008) Generation interval contraction and epidemic data analysis. Math Biosci 213 1 71–9. 18394654 10.1016/j.mbs.2008.02.007 PMC2365921

[43] de la Rua-Domenech R, Goodchild AT, Vordermeier HM, Hewinson RG, Christiansen KH, et al (2006) Ante mortem diagnosis of tuberculosis in cattle: a review of the tuberculin tests, gamma-interferon assay and other ancillary diagnostic techniques. Research in Veterinary Science 81: 190–210. 16513150 10.1016/j.rvsc.2005.11.005

[44] Thom ML, Hope JC, McAulay M, Villarreal-Ramos B, Coffey TJ, et al (2006) The effect of tuberculin testing on the development of cell-mediated immune responses during *Mycobacterium bovis* infection. Vet Immunol Immunopathol 114: 25–36. 16904754 10.1016/j.vetimm.2006.07.001

[45] Neill S, Hanna J, Mackie D, Bryson T (1992) Isolation of *Mycobacterium bovis* from the respiratory tracts of skin test-negative cattle. Vet Rec 131: 45–47. 1441161 10.1136/vr.131.3.45

[46] Sedgewick R (2001) Algorithms in C++. Indianapolis, Indiana: Addison Wesley.

