# Supplementary figures and images for: Whole Genome Sequencing Reveals Local Transmission Patterns of Mycobacterium bovis in Sympatric Cattle and Badger Populations
Source: PLoS Pathog. 2012 Nov 29;8(11):e1003008. doi: 10.1371/journal.ppat.1003008 (PMC3510252; doi:10.1371/journal.ppat.1003008)

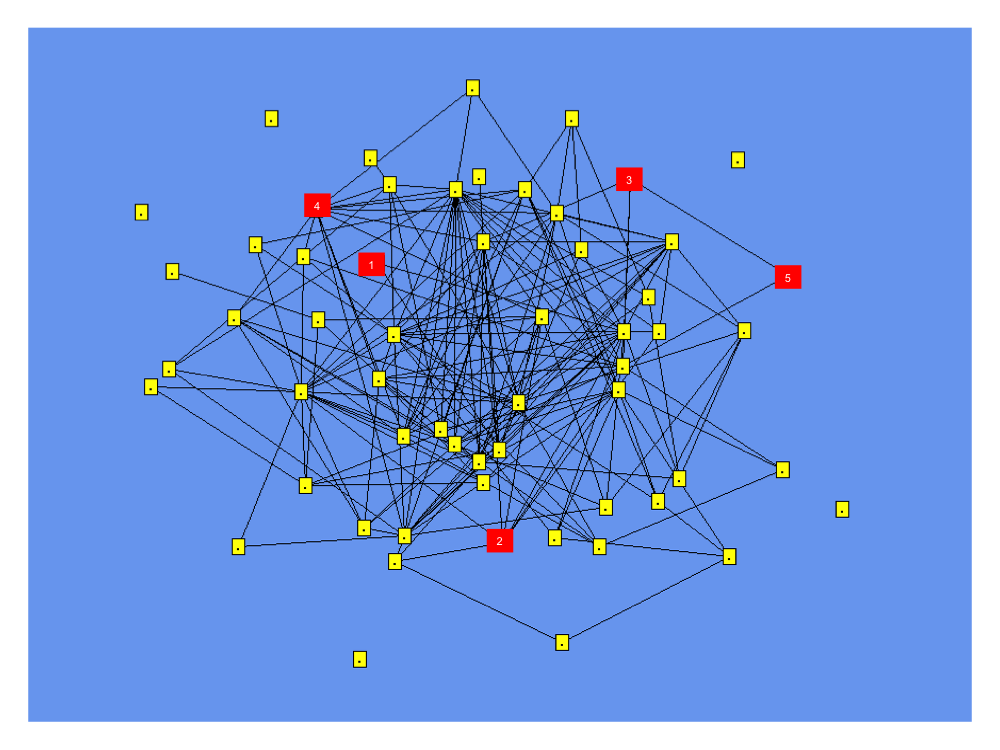

Supplement: Figure S3 — Network of contact via cattle movements amongst all cattle herds in Northern Ireland where breakdowns due to VNTR type 10 have been identified. Herds from which sequenced isolates were derived are indicated in red and number as in Figure 1. All other herds in yellow. (TIF) [file ppat.1003008.s003.tif]

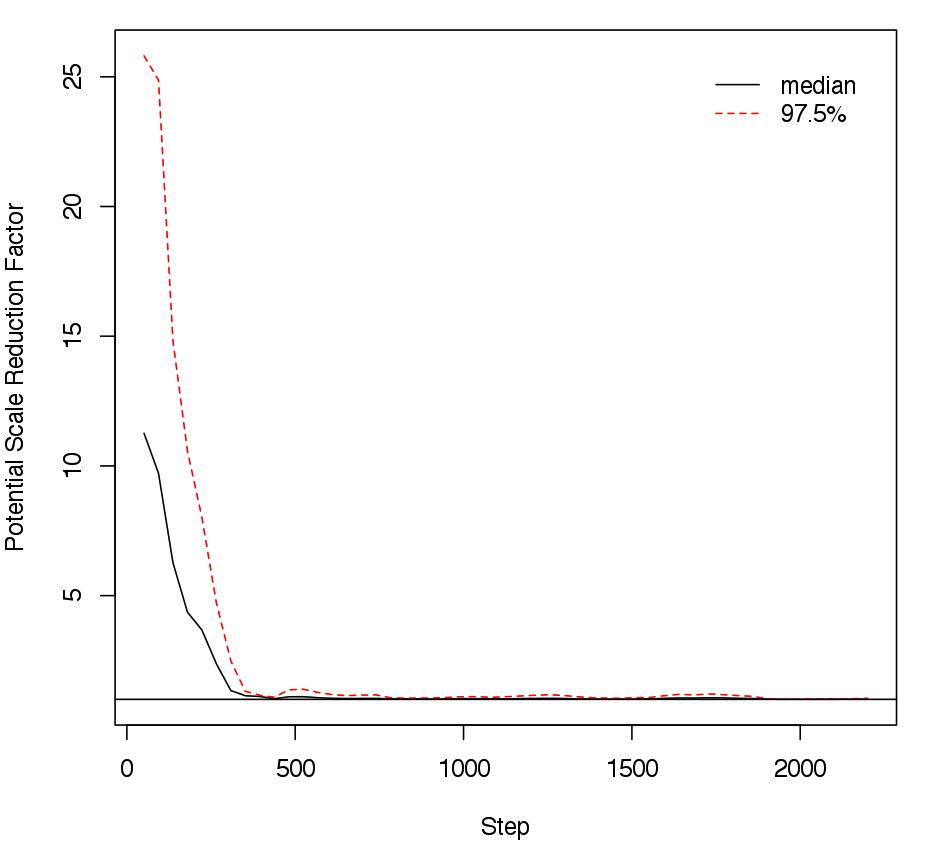

Supplement: Figure S4 — Evolution of the Gelman-Rubin shrink factor for the Markov Chain Monte Carlo chains (burn-in period removed). Each chain (6 in total) was started at a different point in the parameter space of the model. At each step in the chain we perturbed the set of parameters to make the next step and if accepted, calculate the log-likelihood for the model. The potential scale reduction factor is calculated as <1.01 after a long burn in phase indicating convergence has been reached. (TIF) [file ppat.1003008.s004.tif]

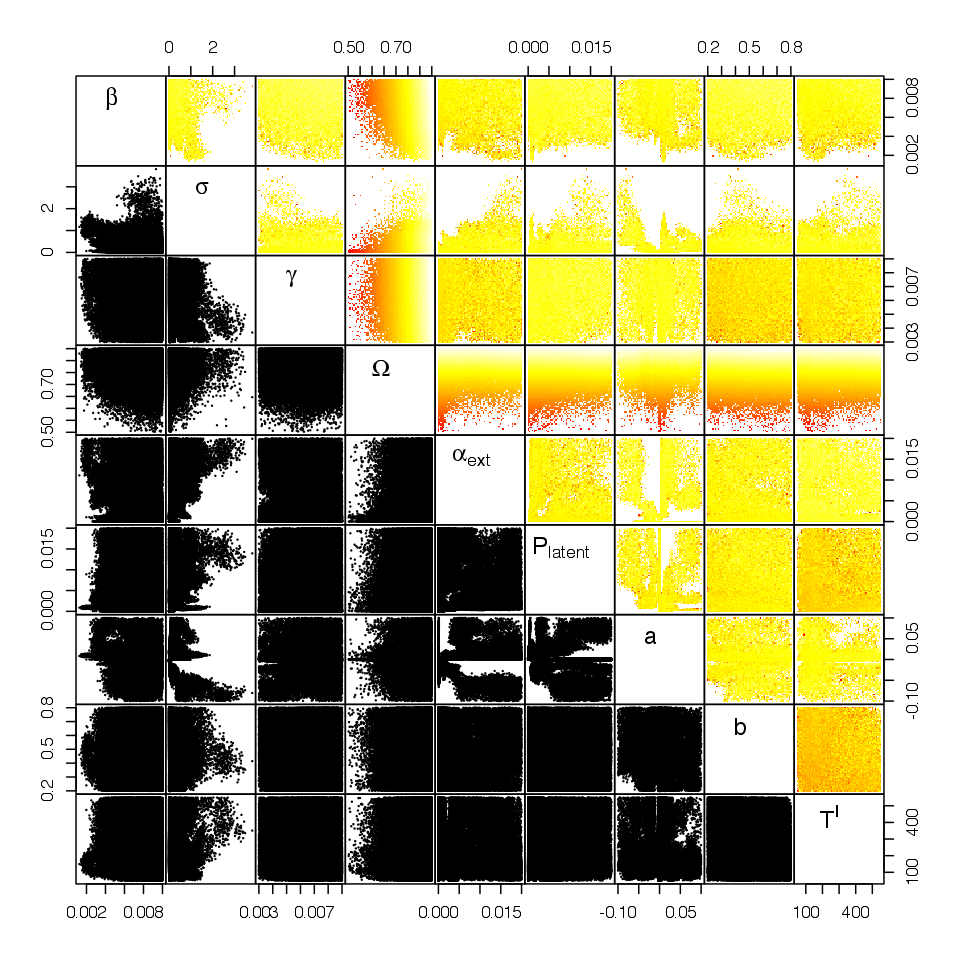

Supplement: Figure S6 — Distribution of the samples taken in the Markov Chain. The lower panel shows all the sampled points of the Markov Chain and the upper is colour coded with the lighter colours denoting those samples corresponding to a higher likelihood. The clumping that is observed in the sampling regime for some parameters is due to the convergence of the chains. Here are the transition rates from the susceptible to exposed, exposed to test sensitive and test sensitive to infectious states respectively, are the external and internal (latently infected animals within the herd) reservoir terms respectively, TI is the length of the infectious stage and a and b inform the probability that a reactor animal was infectious (rather than test sensitive) at the time of a positive test, according to the form , The priors used can be seen from the limits of the sampled points, in each case we used uniform priors over these limits. The parameter L is the maximum length of the infectious period (in days). The priors used for correspond to stages with lengths 1–110 days and 120–280 days respectively, and for , 0.50–0.85. (PNG) [file ppat.1003008.s006.png]

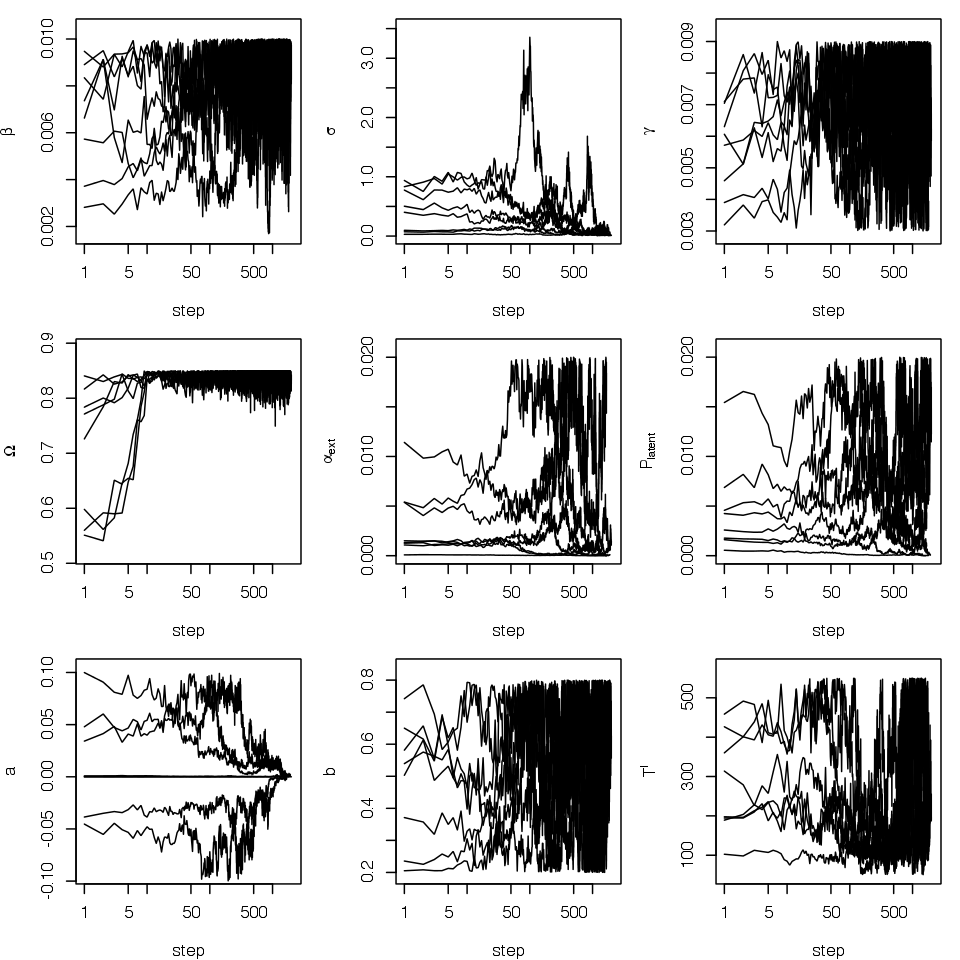

Supplement: Figure S7 — Trace of the parameters of the model. Illustrated are the traces of the parameters used in the model. Convergence towards posterior values in all parameters is observed from the dispersed starting points. Here are the transition rates from the susceptible to exposed, exposed to test sensitive and test sensitive to infectious states respectively, are the external and internal (latently infected animals within the herd) reservoir terms respectively, TI is the length of the infectious stage and a and b inform the probability that a reactor animal was infectious (rather than test sensitive) at the time of a positive test, according to the form , is the sensitivity of the routine herd test applied to each animal. (PNG) [file ppat.1003008.s007.png]
