# Supplementary material for: Whole Genome Sequencing Reveals Local Transmission Patterns of Mycobacterium bovis in Sympatric Cattle and Badger Populations
Source: PLoS Pathog. 2012 Nov 29;8(11):e1003008. doi: 10.1371/journal.ppat.1003008 (PMC3510252; doi:10.1371/journal.ppat.1003008)
Supplement: Text S1 — Supporting Material and Methods: Examining the reliability of SNP calls among the 31 VNTR-10 isolates. (DOC) [file ppat.1003008.s008.doc]

Biek et al - Whole genome sequencing reveals local transmission patterns of *Mycobacterium bovis* in sympatric cattle and badger populations.

**Supplementary materials**

Examining the reliability of SNP calls among the 31 VNTR-10 isolates

To find regions of the bTB genome with high similarity to other regions (which could lead to unreliable mapping), we generated sequences of 55bp (average read length after trimming) across the whole bTB genome. These 55bp sequences were generated every 5bp across the genome, meaning that each base in the genome was covered 11 times (55/5) with the exception of the first and last 55bp in the bTB genome. Each 55bp sequence was then BLASTed (Basic Local Alignment Search Tool) back against the bTB genome and the resulting BLAST hits were filtered using an E-value cutoff of 1e-10, with the number of BLAST hits for each base in the bTB genome tallied. In total, 6.3% bases in the bTB genome received more than the expected 11 BLAST hits, including 4 of the SNPs identified in our analysis – 2 of which only received 1 additional BLAST hit with a number of mismatches. Given that we discounted reads with more than 10% mismatches in the assembly stage, we further filtered the BLAST hits to only consider those with less than 6 mismatches. This further filtering left only 2 SNPs as potentially problematic with 1 to 2 more BLAST hits than expected; both of these SNPS are unique to different individual cattle. Given the small number, these 2 SNPs were then examined manually to see if they were valid. The first SNP, at position 105,516 has high coverage, a high SNP rate, and is the very last base of a perfect repeat region, reads mapped to this position were examined and found to cover the SNP both upstream (into the repeat region) and downstream (into a non-repeat region). Furthermore, our raw read data is paired-end with paired reads mapping ~350bp downstream into regions of no BLAST sequence similarity. The second SNP at position 3,983,266 shows similarity to another region of the bTB genome with BLAST hits of between 3 and 5 mismatches. Given the non-prefect BLAST hits the aligner would favour the correct region first, and the SNP is further validated by our paired end data which maps ~350bp downstream to regions of no BLAST sequence similarity. Overall, the data suggests that these 2 SNPs are valid and could be called correctly.

**Table S1. Assembly statistics for sequenced isolates.**

| **Isolate** | **No. of reads**^a^ | **Average read depth** | **% genome coverage**^b^ |
| --- | --- | --- | --- |
| Badger_LN_2004 | 5,333,897 | 70 | 99.98 |
| Badger_LN_2005 | 6,723,859 | 88 | 99.97 |
| Badger_P&P_2005 | 5,798,179 | 76 | 99.98 |
| Badger_P&P2006 | 4,540,885 | 60 | 99.97 |
| Badger_Kid_2007 | 4,901,155 | 65 | 99.90 |
| Herd1_1999 | 4,676,842 | 61 | 99.90 |
| Herd1_2004 | 7,386,456 | 97 | 99.95 |
| Herd2_1999 | 6,514,971 | 86 | 99.97 |
| Herd3_1999 | 6,666,997 | 87 | 99.96 |
| Herd3_2004 | 4,568,311 | 60 | 99.90 |
| Herd3_A_2007 | 7,052,817 | 94 | 99.97 |
| Herd3_B_2007 | 8,449,703 | 111 | 99.97 |
| Herd3_C_2007 | 5,918,914 | 78 | 99.90 |
| Herd3_D_2007 | 7,431,391 | 98 | 99.96 |
| Herd3_A_2010 | 6,710,423 | 89 | 99.96 |
| Herd3_B_2010 | 5,254,443 | 70 | 99.90 |
| Herd3_C_2010 | 4,969,431 | 65 | 99.90 |
| Herd3_D_2010 | 4,645,856 | 61 | 99.90 |
| Herd3_E_2010 | 6,235,768 | 81 | 99.97 |
| Herd3_F_2010 | 6,386,719 | 85 | 99.97 |
| Herd4_2003 | 8,464,279 | 111 | 99.96 |
| Herd5_A_2008 | 8,331,674 | 112 | 99.96 |
| Herd5_B_2008 | 7,951,564 | 106 | 99.97 |
| Herd5_C_2008 | 6,175,874 | 82 | 99.96 |
| Herd5_D_2008 | 6,365,849 | 85 | 99.90 |
| Herd5_A_2010 | 6,270,295 | 84 | 99.90 |
| Herd5_B_2010 | 7,419,002 | 99 | 99.96 |
| Herd5_C_2010 | 7,129,409 | 95 | 99.98 |
| Herd5_D_2010 | 7,226,702 | 97 | 99.97 |
| Herd5_E_2010 | 5,134,326 | 69 | 99.90 |
| Herd5_F_2010 | 5,803,977 | 78 | 99.90 |

^a^ Number of successfully assembled, trimmed paired-end Illumina 70bp reads (70bp)

^b^ Relative to reference genome AF2122/97 (Garnier et al. 2003)

^c^ two isolates taken from different tissues of the same badger

Tissue abbreviations for badger samples: LN = lymph node; P&P = pleura and pneuma; Kid = kidney

| Reference position | 105166 | 183046 | 220951 | 221272 | 221697 | 221735 | 221927 | 257457 | 361030 | 479247 | 714709 | 740910 | 911427 | 913649 | 1062705 | 1549744 | 1622098 | 1715139 | 1804317 | 1840942 |
| --- | --- | --- | --- | --- | --- | --- | --- | --- | --- | --- | --- | --- | --- | --- | --- | --- | --- | --- | --- | --- |
| gene | hypoth protein | fadE2 | ilvD | ilvD | ilvD | ilvD | non coding | fadD4 | sulfatase | IpqK | galK | mmaA1 | hypoth protein | phoT | sucC | pyrR | zwf2 | fadD24 | hypoth protein | non coding |
| aa change | Phe-Ser | synonym. | San-Lys | synonym. | Arg-Leu | Phe-Val | - | Ala-Thr | Pro-Ser | frameshift | Val-Met | synonym | Leu-Phe | Val-Ile | Glu-Ala | Val-Met | His-Tyr | Arg-His | Ala-Val | - |
| Reference (AF | A | G | T | C | G | T | G | G | C | T | G | C | C | G | A | G | C | A | C | T |
| Badger_2004 | . | . | . | . | . | . | A (77x, 99%) | . | . | . | . | . | . | . | . | . | . | . | T (49x, 100%) | . |
| Badger_2006 | . | . | . | . | . | . | . | . | . | . | . | . | . | . | . | A (40x, 100%) | . | . | T (38x, 100%) | . |
| Badger_2007 | . | . | . | . | . | . | . | . | . | . | . | T (94x, 100%) | . | . | . | . | . | G (58x, 98%) | . | . |
| Badger_LN_2005 | . | A (66x, 99%) | . | . | . | . | . | . | . | . | . | . | . | . | . | . | . | . | T (69x, 100%) | . |
| Badger_P&P_2005 | . | . | . | . | T (90x, 100%) | . | . | . | . | . | . | . | . | . | . | . | . | . | T (63x, 100%) | C (71x, 100%) |
| Herd1_1999 | . | . | . | . | . | . | . | . | . | . | . | T (134x, 100%) | . | . | . | . | . | . | . | . |
| Herd1_2004 | . | . | . | . | . | . | . | . | . | . | . | T (124x, 100%) | . | . | . | . | . | . | . | . |
| Herd2_1999 | . | . | . | . | . | . | A (104x, 100%) | . | . | . | A (56x, 100%) | . | . | . | . | . | . | . | . | . |
| Herd3_1999 | . | . | . | . | . | . | . | . | . | . | . | . | . | . | . | . | . | . | T (50x, 100%) | . |
| Herd3_2004 | . | . | . | . | . | . | . | . | . | - (100x, 83%) | . | T (132x, 100%) | . | . | C (118x, 99%) | . | . | G (97x, 100%) | . | . |
| Herd3_A_2007 | . | . | . | . | . | . | . | . | . | . | . | T (162x, 99%) | . | . | . | . | . | G (114x, 100%) | . | . |
| Herd3_A_2010 | . | . | A (53x, 100%) | . | . | . | . | . | . | . | . | T (111x, 99%) | . | . | . | . | T (66x, 100%) | G (72x, 100%) | . | . |
| Herd3_B_2007 | G (105x, 92%) | . | . | . | . | . | . | . | . | . | . | T (109x, 100%) | . | . | . | . | . | G (52x, 100%) | . | . |
| Herd3_B_2010 | . | . | . | . | . | . | . | . | . | . | . | T (97x, 100%) | . | . | . | . | . | G (56x, 100%) | . | . |
| Herd3_C_2007 | . | . | . | . | . | . | . | . | . | . | . | T (158x, 100%) | . | . | . | . | . | G (95x, 100%) | . | . |
| Herd3_C_2010 | . | . | . | . | . | . | . | . | . | . | . | T (96x, 100%) | . | . | . | . | . | G ( 62x, 98%) | . | . |
| Herd3_D_2007 | . | . | . | . | . | . | . | . | . | . | . | T (140x, 100%) | . | . | . | . | . | G (94x, 100%) | . | . |
| Herd3_D_2010 | . | . | . | G (92x, 99%) | . | . | . | . | . | . | . | T (108x, 100%) | . | . | . | . | . | G (89x, 100%) | . | . |
| Herd3_E_2010 | . | . | . | . | . | . | . | . | . | . | . | T (135x, 100%) | . | . | . | . | . | G (63x, 100%) | . | . |
| Herd3_F_2010 | . | . | . | . | . | . | . | . | . | . | . | . | . | . | . | . | . | . | . | . |
| Herd4_2003 | . | . | . | . | . | . | . | . | . | . | . | T (161x, 100%) | . | A (97x, 80%) | . | . | . | G (107x, 100%) | . | . |
| Herd5_A_2008 | . | . | . | . | . | . | . | . | T (139x, 98%) | . | . | T (133x, 100%) | . | . | . | . | . | G (103x, 100%) | . | . |
| Herd5_A_2010 | . | . | . | . | . | G (136x, 79%) | . | . | . | . | . | T (97x, 100%) | . | . | . | . | . | G (70x, 100%) | . | . |
| Herd5_B_2008 | . | . | . | . | . | . | . | A (109x, 99%) | . | . | . | T (188x, 100%) | T (160x, 100%) | . | . | . | . | G (91x, 100%) | . | . |
| Herd5_B_2010 | . | . | . | . | . | . | . | . | . | . | . | T (140x, 100%) | . | . | . | . | . | G (82x, 97%) | . | . |
| Herd5_C_2008 | . | . | . | . | . | . | . | . | . | . | . | T (111x, 100%) | . | . | . | . | . | G (85x, 100%) | . | . |
| Herd5_C_2010 | . | . | . | . | . | . | A (124x, 95%) | . | . | . | . | T (123x, 100%) | . | . | . | . | . | G (92x, 100%) | . | . |
| Herd5_D_2008 | . | . | . | . | . | . | . | . | . | . | . | T (134x, 100%) | . | . | . | . | . | G (67x, 100%) | . | . |
| Herd5_E_2010 | . | . | . | . | . | . | A (92, 64%) | . | . | . | . | T (104, 100%) | . | . | . | . | . | G (63, 100%) | . | . |
| Herd5_F_2010 | . | . | . | . | . | . | . | . | . | . | . | T (127, 100%) | . | . | . | . | . | G (67, 100%) | . | . |

Table S2. Genomic position and support for 39 SNPs detected among the 31 *M. bovis* genomes. Values in brackets represent depth of coverage and percent support out of all reads.

Table S2 (cont)

| Reference position | 1931809 | 2093709 | 2128519 | 2475044 | 2483502 | 2513425 | 2560919 | 2560919 | 2702921 | 3374217 | 3376506 | | 3584771 | 3642915 | 3715671 | 3968499 | 3968577 | 3979397 | 3983266 | 4299547 |
| --- | --- | --- | --- | --- | --- | --- | --- | --- | --- | --- | --- | --- | --- | --- | --- | --- | --- | --- | --- | --- |
| gene | non coding | Oxido-reductase | non coding | exported protease | hypoth protein | hypoth protein | hypoth protein | hypoth protein | hypoth protein | dinP | cyp136 | TetR-type transcript regulator | | glpD2 | PPE56b | Putative transcript factor | Putative transcript factor | hypoth protein | clpC | hypoth protein |
| AA change | - | synonym | - | Val-Gly | His-Tyr | Ala-Val | Gly-Asn | Gly-Asn | Val-Ala | Ala-Thr | Val-Ile | synonym | | Gly-Ser | Pro-Leu | synonym | Asp-Glu | Gly-Asp | Phe-Tyr | Phe-Ser |
| Reference sequence | A | C | T | T | C | C | G | G | T | G | G | C | | G | G | G | C | G | T | T |
| Badger_2004 | . | . | . | . | . | . | . | . | . | . | A (75, 100%) | . | | . | . | . | . | . | . | . |
| Badger_2006 | . | T (45, 100%) | . | . | . | . | . | . | . | A (72, 97%) | A (56, 100% | . | | . | . | . | . | A (57, 100%) | . | . |
| Badger_2007 | . | . | . | . | . | C (50, 100%) | . | . | . | . | . | . | | . | . | . | . | . | . | . |
| Badger_LN_2005 | . | . | . | . | . | . | . | . | . | . | A (92, 100%) | . | | . | . | . | A (119, 98%) | . | . | . |
| Badger_P&P_2005 | . | . | G (84, 100%) | . | . | . | . | . | . | . | A (78, 98%) | . | | . | . | . | . | . | . | . |
| Herd1_1999 | . | . | . | G (102, 100%) | . | . | . | . | . | . | . | . | | . | . | . | . | . | . | . |
| Herd1_2004 | . | . | . | . | . | . | . | . | . | . | . | G (44, 98%) | | . | . | . | . | . | . | . |
| Herd2_1999 | . | . | . | . | . | . | . | . | C (78,100%) | . | . | . | | . | A (91, 99%) | . | . | . | . | . |
| Herd3_1999 | . | . | . | . | . | . | . | . | . | . | A (56, 100%) | . | | . | . | . | . | . | . | . |
| Herd3_2004 | . | . | . | . | . | C (68, 100%) | . | . | . | . | . | . | | . | . | . | . | . | . | . |
| Herd3_A_2007 | . | . | . | . | . | C (97, 99.0%) | . | . | . | . | . | . | | . | . | . | . | . | . | . |
| Herd3_A_2010 | . | . | . | . | . | C (52, 100%) | . | . | . | . | . | . | | . | . | . | . | . | . | . |
| Herd3_B_2007 | . | . | . | . | . | C (58, 98%) | . | . | . | . | . | . | | . | . | . | . | . | . | . |
| Herd3_B_2010 | . | . | . | . | T (45, 100%) | C (60, 100%) | . | . | . | . | . | . | | . | . | . | . | . | . | . |
| Herd3_C_2007 | . | . | . | . | . | C (87, 99%) | . | . | . | . | . | . | | . | . | . | . | . | . | . |
| Herd3_C_2010 | . | . | . | . | . | C (58, 100%) | . | . | . | . | . | . | | . | . | . | . | . | . | . |
| Herd3_D_2007 | . | . | . | . | . | C (63, 97%) | . | . | . | . | . | . | | . | . | . | . | . | . | . |
| Herd3_D_2010 | . | . | . | . | . | C (72, 96%) | . | . | . | . | . | . | | A (76, 100%) | . | . | . | . | . | . |
| Herd3_E_2010 | . | . | . | . | . | C (74, 100%) | . | . | . | . | . | . | | . | . | . | . | . | . | . |
| Herd3_F_2010 | G (73, 86%) | . | . | . | . | . | . | . | . | . | A (105, 98%) | . | | . | . | . | . | . | . | . |
| Herd4_2003 | . | . | . | . | . | . | . | . | . | . | . | . | | . | . | . | . | . | . | . |
| Herd5_A_2008 | . | . | . | . | . | C (86, 100%) | . | . | . | . | . | . | | . | . | . | . | . | A (120, 98%) | . |
| Herd5_A_2010 | . | . | . | . | . | C (58, 100%) | . | . | . | . | . | . | | . | . | . | . | . | . | . |
| Herd5_B_2008 | . | . | . | . | . | C (86, 100%) | . | . | . | . | . | . | | . | . | A (173, 98%) | . | . | . | . |
| Herd5_B_2010 | . | . | . | . | . | C (86, 91%) | A (87, 99%) | A (87, 99%) | . | . | . | . | | . | . | . | . | . | . | . |
| Herd5_C_2008 | . | . | . | . | . | C (66, 100%) | . | . | . | . | . | . | | . | . | . | . | . | . | . |
| Herd5_C_2010 | . | . | . | . | . | C (77, 100%) | . | . | . | . | . | . | | . | . | . | . | . | . | C (81, 100%) |
| Herd5_D_2008 | . | . | . | . | . | C (63, 100%) | . | . | . | . | . | . | | . | . | . | . | . | . | . |
| Herd5_E_2010 | . | . | . | . | . | C (99, 100%) | . | . | . | . | . | . | | . | . | . | . | . | . | C (87, 100%) |
| Herd5_F_2010 | . | . | . | . | . | C (70, 100%) | . | . | . | . | . | . | | . | . | . | . | . | . | . |

**Figure S1: Maximum likelihood tree of 31 *M. bovis* genomes based on 38 concatenated SNPs**. Tree search was conducted in phyml under a Juke-Cantor model and reference sequence AF2122/97 was used as an outgroup (removed for clarity) to establish the root node.

**Figure S2. Accumulation of genetic changes in *M. bovis* genomes through time.** Genetic divergence of 31 cattle and badger isolates from the root node (measured in substitutions per genome) increases with sampling date, consistent with clock-like evolution. The estimated slope corresponds to an evolutionary rate of 3.40 (CI: 0.87-5.93) x 10-8 substitutions per site per year. To improve visibility, points were jittered randomly.


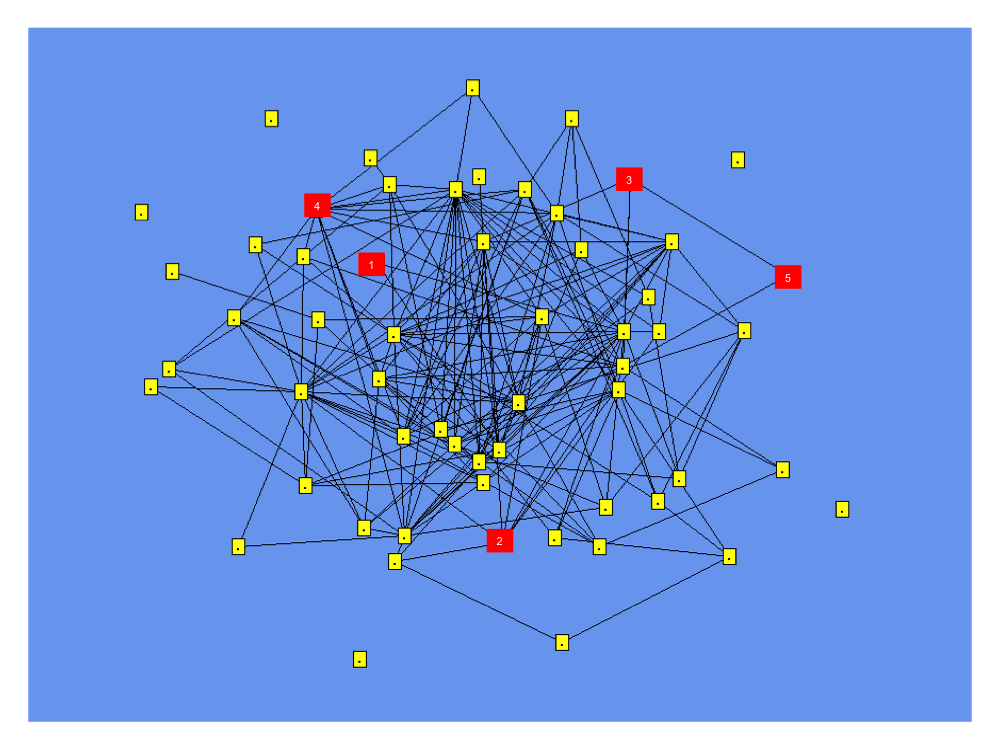


**Figure S3. Network of contact via cattle movements.** The network includes all cattle herds in Northern Ireland where breakdowns due to VNTR type 10 have been identified. Herds from which sequenced isolates were derived are indicated in red and number as in figure 1. All other herds in yellow.

**
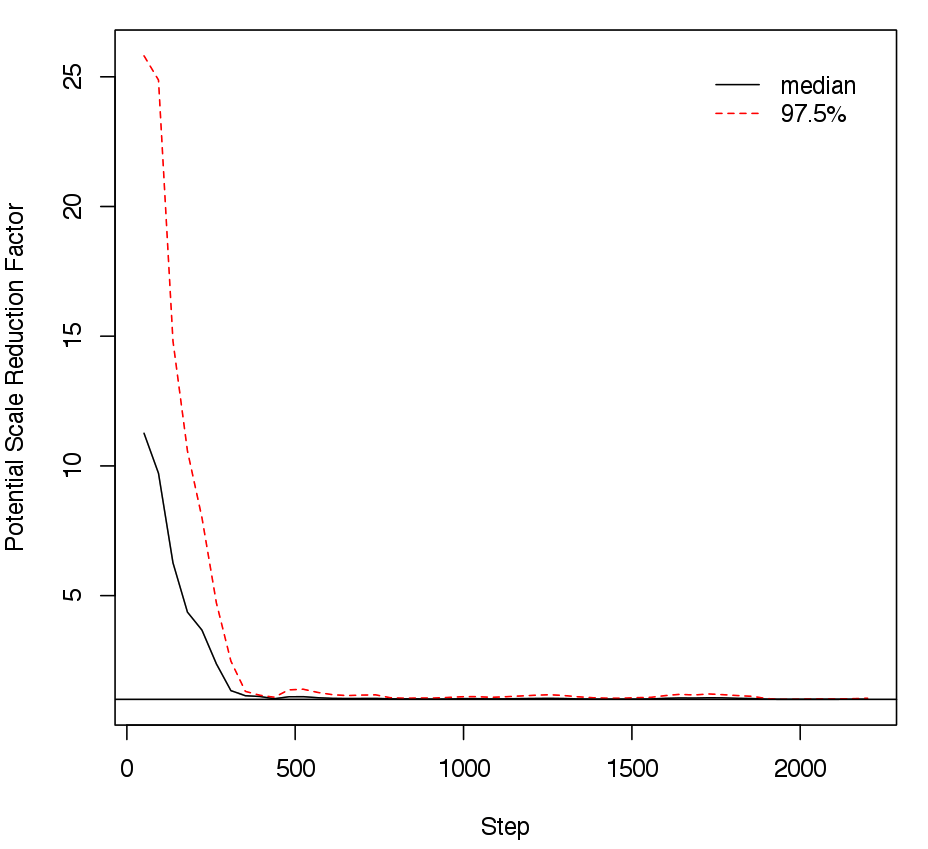
**

**Fig S4 : Evolution of the Gelman-Rubin shrink factor for the Markov Chain Monte Carlo chains after the burn-in period has been removed.** Each chain (6 in total) was started at a different point in the parameter space of the model**.** At each step in the chain we perturbed the set of parameters to make the next step and if accepted, calculate the log-likelihood for the model. The potential scale reduction factor is calculated as < 1.01 after a long burn in phase indicating convergence has been reached.


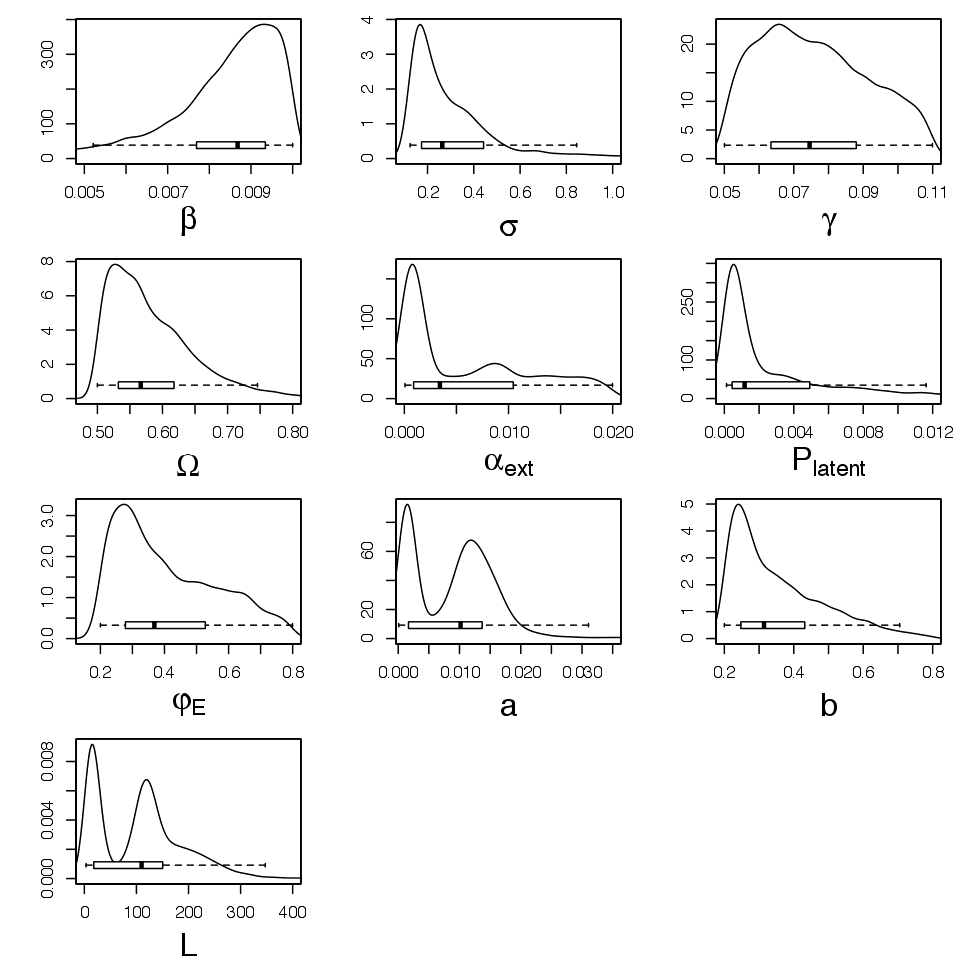


**Figure S5: Posterior kernel density estimates.** Illustrated are the distributions obtained from the MCMC chains after the burn-in period was removed. Convergence towards optimum values in all parameters is observed, with considerable mixing of the parameters. Here are the transition rates from the susceptible to exposed, exposed to test sensitive and test sensitive to infectious states respectively, are the external and internal (latently infected animals within the herd) reservoir terms respectively, T^I^ is the length of the infectious stage and *a* and *b* inform the probability that a reactor animal was infectious (rather than test sensitive) at the time of a positive test, according to the form . Mean posterior values with 95% credible intervals ($\beta$* *= 3.52 [2.27, 4.77]x10^-3^ fortnight^-1^ $s$* * = 0.387 [0.382, 0.392] fortnight^-1^, $g$= 0.266 [0.262, 0.270] fortnight^-1^, $\alpha_{\text{ext}}$= 0.633 [0.631, 0.635] fortnight^-1^, *P_latent_* = 1.010 [0.600, 1.430]x10^-4^ fortnight^-1^, *a* = 0, *b* = 0.0860 [0.0837, 0.0884], *T^I^* = 71.6 [68.9, 74.3] days. See **Figure S6** for the distribution of all the points sampled.


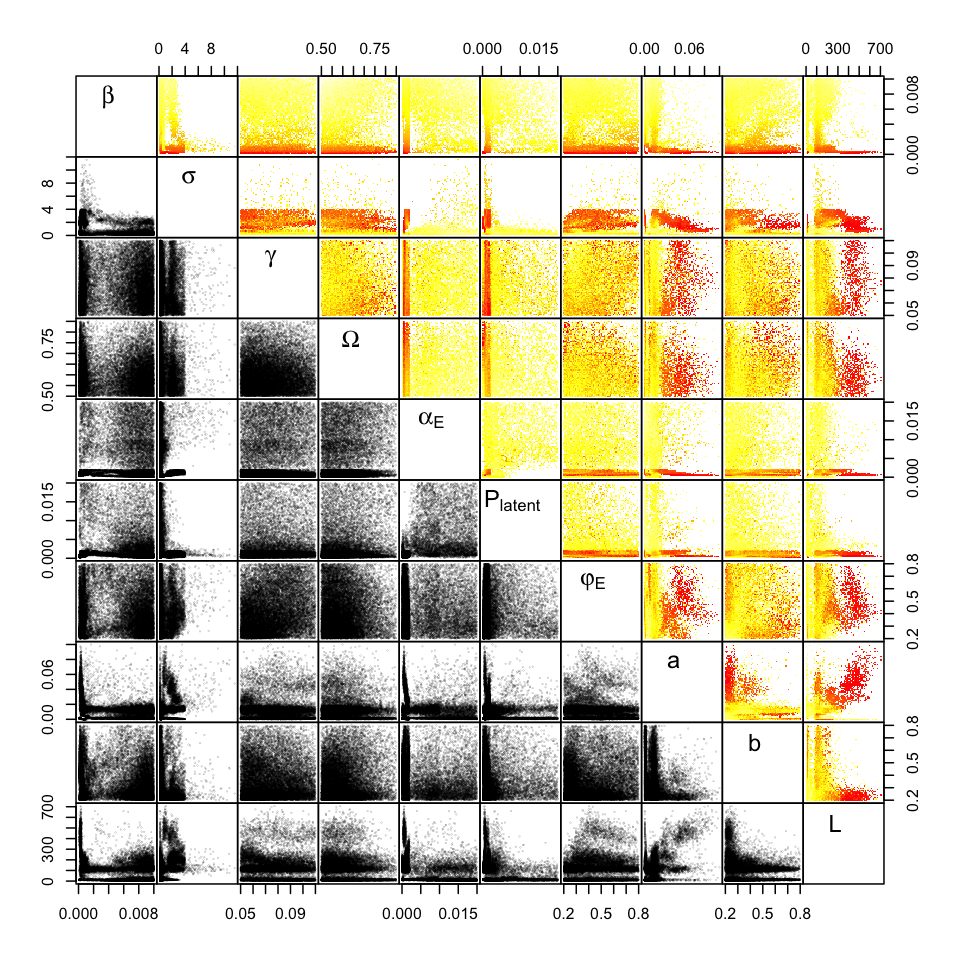


**Figure S6: Distribution of the samples taken in the Markov Chain.** The lower panel shows all the sampled points of the Markov Chain and the upper is colour coded with the lighter colours denoting those samples corresponding to a higher likelihood. The clumping that is observed in the sampling regime for some parameters is due to the convergence of the chains. Here $\beta,\sigma,\gamma$are the transition rates from the susceptible to exposed, exposed to test sensitive and test sensitive to infectious states respectively, are the external and internal (latently infected animals within the herd) reservoir terms respectively, T^I^ is the length of the infectious stage and *a* and *b* inform the probability that a reactor animal was infectious (rather than test sensitive) at the time of a positive test, according to the form , The priors used can be seen from the limits of the sampled points, in each case we used uniform priors over these limits. The parameter *L* is the maximum length of the infectious period (in days). The priors used for correspond to stages with lengths 1-110 days and 120-280 days respectively, and for $\Omega$, 0.50-0.85.


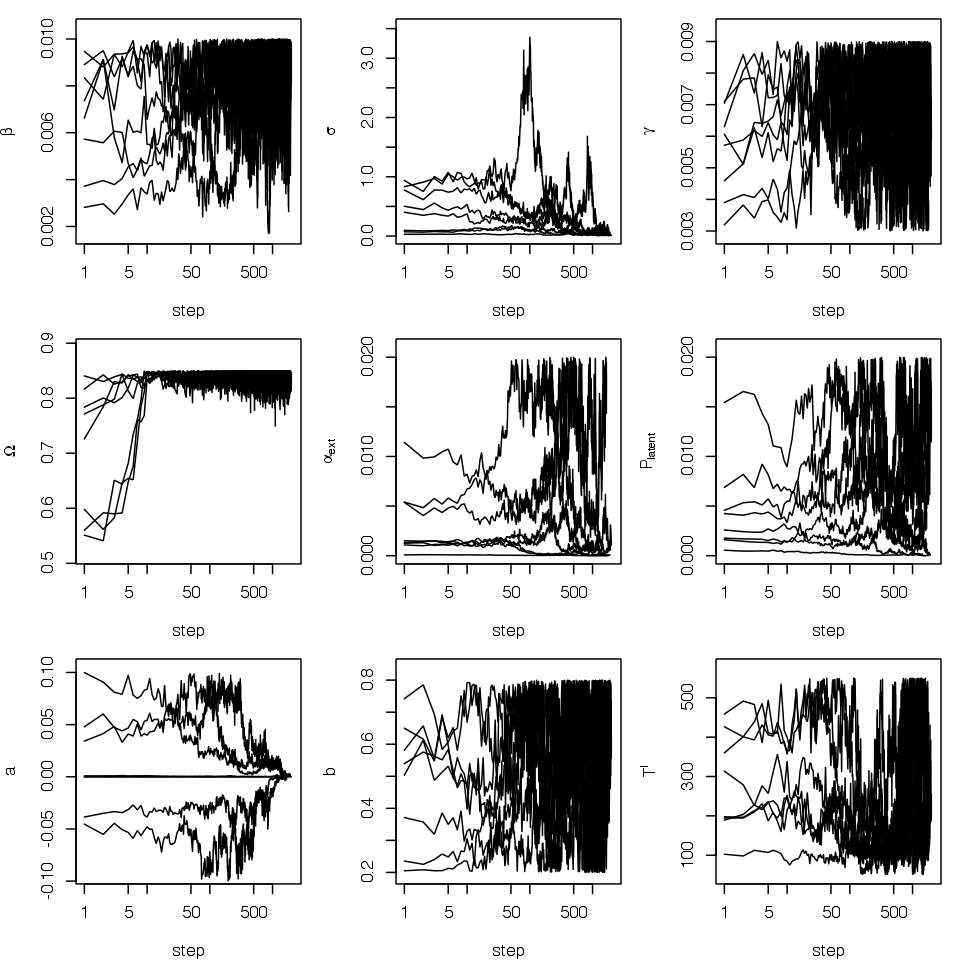


**Figure S7: Trace of the parameters of the model.** Illustrated are the traces of the parameters used in the model. Convergence towards posterior values in all parameters is observed from the dispersed starting points. Here $\beta,\sigma,\gamma$are the transition rates from the susceptible to exposed, exposed to test sensitive and test sensitive to infectious states respectively, are the external and internal (latently infected animals within the herd) reservoir terms respectively, T^I^ is the length of the infectious stage and *a* and *b* inform the probability that a reactor animal was infectious (rather than test sensitive) at the time of a positive test, according to the form , is the sensitivity of the routine herd test applied to each animal.
